# The liverwort oil body is formed by redirection of the secretory pathway

**DOI:** 10.1038/s41467-020-19978-1

**Published:** 2020-12-01

**Authors:** Takehiko Kanazawa, Hatsune Morinaka, Kazuo Ebine, Takashi L. Shimada, Sakiko Ishida, Naoki Minamino, Katsushi Yamaguchi, Shuji Shigenobu, Takayuki Kohchi, Akihiko Nakano, Takashi Ueda

**Affiliations:** 1 Division of Cellular Dynamics, National Institute for Basic Biology, Nishigonaka 38, Myodaiji, Okazaki, Aichi 444-8585 Japan; 2 The Department of Basic Biology, SOKENDAI (The Graduate University for Advanced Studies), Nishigonaka 38, Myodaiji, Okazaki, Aichi 444-8585 Japan; 3 Department of Biological Sciences, Graduate School of Sciences, The University of Tokyo, 7-3-1 Hongo, Bunkyo-ku, Tokyo, 113-0033 Japan; 4 Department of Applied Biological Chemistry, Graduate School of Horticulture, Chiba University, 648 Matsudo, Matsudo, Chiba Japan; 5 Graduate School of Biostudies, Kyoto University, Kitashirakawa-oiwake-cho, Sakyo-ku, Kyoto, 606-8502 Japan; 6 Functional Genomics Facility, National Institute for Basic Biology (NIBB), Okazaki, Aichi 444-8585 Japan; 7 Live Cell Super-Resolution Imaging Research Team, RIKEN Center for Advanced Photonics, 2-1 Hirosawa, Wako, Saitama, 351-0198 Japan

**Keywords:** Protein trafficking in plants, Cell fate

## Abstract

Eukaryotic cells acquired novel organelles during evolution through mechanisms that remain largely obscure. The existence of the unique oil body compartment is a synapomorphy of liverworts that represents lineage-specific acquisition of this organelle during evolution, although its origin, biogenesis, and physiological function are yet unknown. We find that two paralogous syntaxin-1 homologs in the liverwort *Marchantia polymorpha* are distinctly targeted to forming cell plates and the oil body, suggesting that these structures share some developmental similarity. Oil body formation is regulated by an ERF/AP2-type transcription factor and loss of the oil body increases *M*. *polymorpha* herbivory. These findings highlight a common strategy for the acquisition of organelles with distinct functions in plants, via periodical redirection of the secretory pathway depending on cellular phase transition.

## Abstract (web summary)

Liverworts have a unique oil body organelle unrelated to lipid stores found in other eukaryotes. Here the authors show that oil body formation is analogous to that of cell plates, relying on periodic redirection of the secretory pathway and a syntaxin-1 homolog, and that oil bodies contribute to defence against herbivory.

## Introduction

Eukaryotic cells originated from prokaryotes by expanding their endomembrane network during evolution, with the last eukaryotic common ancestor (LECA) likely possessing a complex set of organelles^1^. New membrane trafficking pathways were added to the LECA endomembrane network, some of which were secondarily lost in a lineage-specific manner, resulting in divergent and organism-specific membrane trafficking systems and organelle compositions of extant eukaryotes^2–4^. Comparative genomics has proposed that the emergence of a novel membrane-bounded organelle was accompanied by the development of a novel membrane trafficking pathway, which was accomplished by expansion and functional differentiation of machinery components through gene duplication followed by neo- and/or subfunctionalization of those machineries by accumulated mutations^5^. For example, coat-protein complexes, RAB GTPases, and SNARE proteins, which act in transport vesicle formation, tethering of the vesicles to target membranes, and membrane fusion between the two membranes, respectively, were shown to expand and functionally differentiate during evolution to develop the specialized membrane trafficking system in each organism^6,7^. However, empirical support for this hypothesis, i.e. the organelle paralogy hypothesis, still remains to be provided.

In the plant lineage, several organelles and organelle functions have been uniquely acquired during evolution. For example, the plant vacuole harbours a unique function that is not shared with the animal lysosome and the yeast vacuole: storage of proteins^8^. This vacuolar function is fulfilled through the plant-unique vacuolar trafficking system, which comprises multiple vacuolar transport pathways involving plant-unique machinery components acquired during plant evolution^9^. The cell plate, which is formed during the mitotic phase to accomplish cytokinesis in land plants, is also a prominent example of plant-specific cellular structures/organelles^10^.

One of the basal-most land plant lineages, liverworts, also possesses a unique organelle, the oil body, the existence of which is a synapomorphy of this lineage. The liverwort oil body contains bioactive compounds, such as sesquiterpenoids and cyclic bisbibenzyl compounds, and is not related to the oil body that stores neutral lipids in storage organs like seeds and fruits (i.e. lipid body or oleosome). About 90% of liverwort species have this organelle; however, its origin, biogenesis, and physiological function remain unclear with controversial origins proposed from microscopic observations^11–15^, although the first description of the liverwort oil body dates back to 1834^16^. Through the systematic analysis of SNARE proteins in the liverwort, *Marchantia polymorpha* (hereafter referred to as Marchantia), we identified an oil body-resident protein, MpSYP12B^17^, which is a homolog of animal syntaxin-1 that acts in the final step of exocytosis. Functions of SYP1 members to which MpSYP12B belongs dramatically diversified during plant evolution, suggesting expanded secretory trafficking systems in plants. Arabidopsis SYP1 consists of three subgroups, SYP11, SYP12, and SYP13. The SYP13 group mediates constitutive secretion, whereas the other groups are involved in plant-specific, higher-ordered functions; for example, KNOLLE (also known as SYP111) is specifically involved in membrane fusion at forming cell plates, and PEN1 (also known as SYP121) is required for intact penetration resistance against powdery mildew fungi and regulation of the ion channel activity^18–23^. Tip growth of root hairs and pollen tubes also involves SYP1 members; distinctive SYP1 members exhibit different expression and localization patterns in tip-growing cells, indicating diverged and specialized functions of SYP1 members in polar secretion in Arabidopsis^20,24–26^.

In this study, for more insight into the functional diversification of SYP1 members in Marchantia and the mechanism of oil body biogenesis, we further characterized four SYP1 members in this organism^17,27^. MpSYP12A and MpSYP12B are distinctly targeted to the forming cell plate and oil body membrane, respectively, representing the cell-phase specific redirection of the secretory pathway. Furthermore, we identified a master transcription factor of oil body formation. Our findings indicate that the cell plate and the oil body are paralogous cellular structures/organelles acquired during plant evolution, and also provide a strong support for the organelle paralogy hypothesis.

## Results

### MpSYP12A is important for cell plate formation

**Fig. 1:**
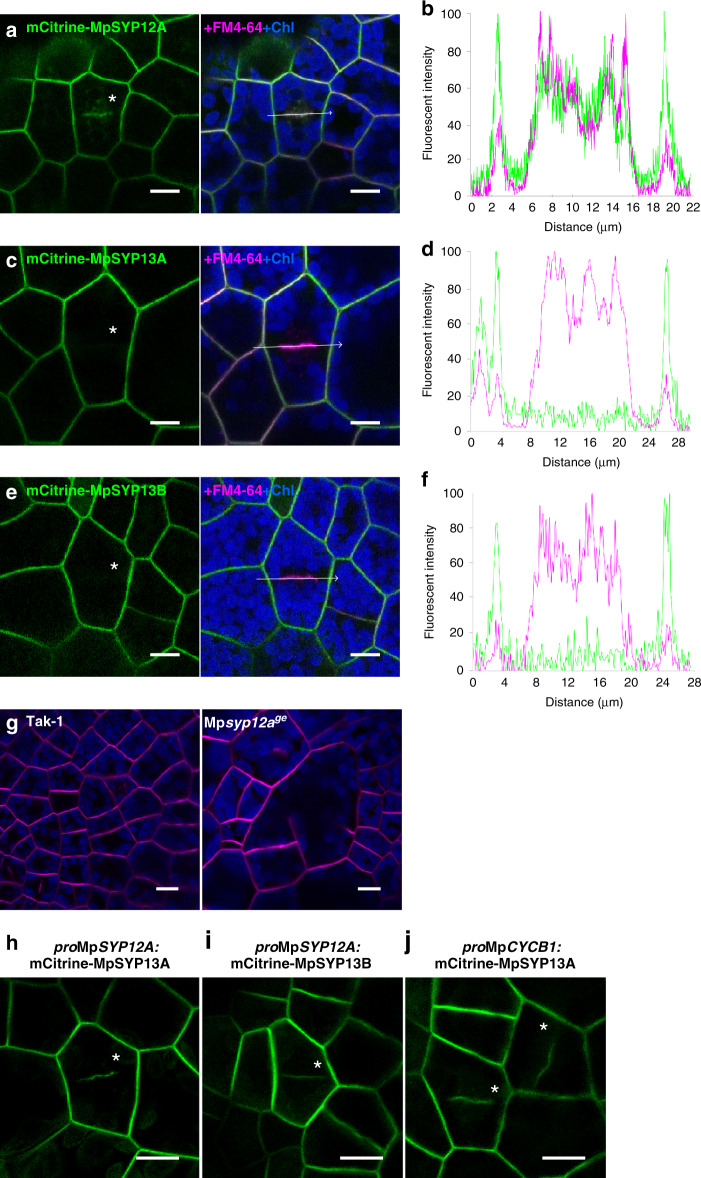
MpSYP12A is important for cell plate formation in *M*. *polymorpha* thallus cells. **a**, **c**, **e** Marchantia thallus cells expressing mCitrine-MpSYP12A (**a**), mCitrine-MpSYP13A (**c**), and mCitrine-MpSYP13B (**e**) stained with FM4-64. **b**, **d**, **f** Line graphs showing relative fluorescence intensities from mCitrine and FM4-64 along the white arrows in **a** (**b**), **c** (**d**), and **e** (**f**). **g** Thallus cells of Tak-1 (left) and a putative knock-out mutant (right) stained with FM4-64. **h**, **i** Dividing thallus cells expressing mCitrine-MpSYP13A (**h**) or mCitrine-MpSYP13B (**i**) driven by the Mp*SYP12A* promoter. **j** Dividing thallus cells expressing mCitrine-MpSYP13A under the regulation of the Mp*CYCB1* promoter. Asterisks indicate forming cell plates. Green, magenta, and blue pseudo-colours indicate fluorescence from mCitrine, FM4-64, and chlorophyll, respectively. Bars = 10 μm.

Our phylogenetic analysis suggested that MpSYP1 members belong to two distinct subgroups: the SYP13 group (MpSYP13A and 13B) and the SYP11/12 group (MpSYP12A and 12B) that contains SYP11 and SYP12 groups in seed plants (Supplementary Fig. 1a)^26^. MpSYP12A, 13A, and 13B were ubiquitously expressed and localized to the plasma membrane (PM) in thalli, whereas MpSYP12B exhibited specific expression in a subpopulation of thallus cells (Supplementary Fig. 1b–e). Using ubiquitously expressed mCitrine-MpSYP1 protein fusion constructs driven by their own regulatory elements, we detected localization of MpSYP12A at forming cell plates associated with the phragmoplast that rapidly stained with FM4-64 in addition to the PM, whereas MpSYP13A, 13B, and 12B were not detected at forming cell plates (Fig. 1a–f, Supplementary Fig. 1f, g). This localization suggested that MpSYP12A could be a functional counterpart of KNOLLE in Arabidopsis, which was further verified genetically.

We tried to generate a complete Mp*syp12a* knockout mutant by genome editing (Supplementary Fig. 1h) but were not successful. Although we could not completely rule out the possibility of impaired functions of other genes due to off-target effects, it was likely because MpS*YP12A* is an essential gene. However, we succeeded in isolating chimeric plants comprising mutated and wild-type cells, in which we frequently observed enlarged cells with cell wall stabs, suggesting that MpSYP12A is required for cell plate formation during cytokinesis similar to the function of KNOLLE (Fig. 1g). The *KNOLLE* promoter is sufficient to target non-cytokinesis-specific SYP132 to forming cell plates^28^. Similarly, mCitrine-tagged MpSYP13A and 13B were localized to cell plates and the PM when expressed under the Mp*SYP12A* promoter (Fig. 1h, i). Furthermore, a similar localization was also observed when mCitrine-MpSYP13 was expressed by the Mp*CYCB1* promoter (Fig. 1j and Supplementary Fig. 1i), indicating that the cell cycle-dependent transcriptional regulation is also critical for cell plate targeting of SYP1 proteins in Marchantia. We also found that clathrin light chain (CLC) tagged with Citrine localized to the PM and *trans*-Golgi network in non-dividing thallus cells, as well as on forming cell plates in Marchantia as reported in other plants (Supplementary Fig. 1j, k)^29–31^. These results strongly suggested that fundamental mechanisms of cell plate formation were conserved during land plant evolution.

### The oil body and plasma membranes share common properties

**Fig. 2:**
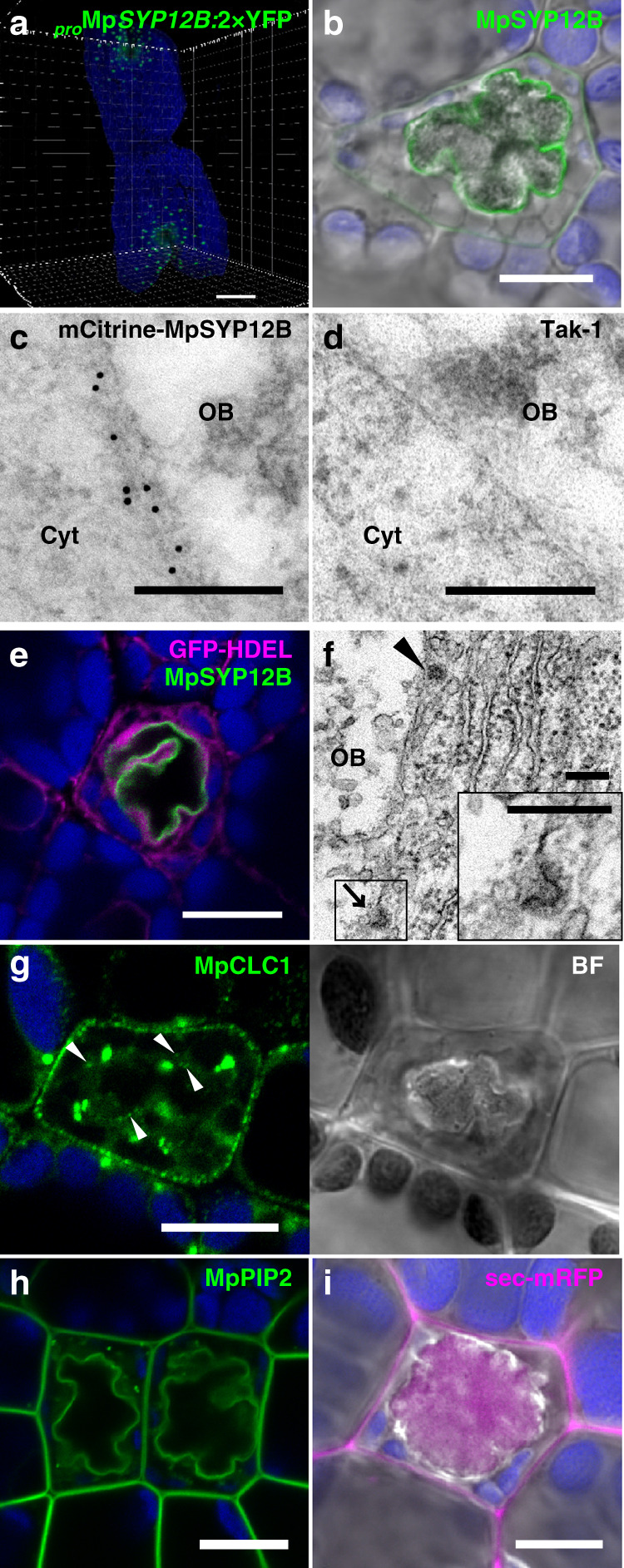
The oil body membrane shares common properties with the plasma membrane. **a** A five-day-old thallus expressing 2×Citrine (YFP) under the regulation of the Mp*SYP12B* promoter observed with a light-sheet microscope. **b** An oil body cell expressing mCitrine-MpSYP12B under its own regulatory elements. The bright-field image was merged. **c**, **d** Immunoelectron micrographs of oil body cells in thalli of Tak-1 with (**c**) or without (**d**) mCitrine-MpSYP12B expression. Cyt, cytosol, and OB, oil body. **e** An oil body cell expressing GFP-HDEL (magenta) and mCitrine-MpSYP12B (green). **f** An electron micrograph of the oil body cell. The arrowhead and arrow indicate a clathrin-coated vesicle and a clathrin-coated pit, respectively. The magnified image of the boxed region is shown in the inset. **g** An oil body cell expressing MpCLC1-Citrine. Arrowheads indicate clathrin-coated vesicles/pits on the oil body membrane. BF, bright-field image. **h** Oil body cells expressing mCitrine-MpPIP2 under its own regulatory elements. **i** An oil body cell expressing sec-mRFP driven by the Mp*EF1α* promoter. The bright-field image was merged. The blue pseudo-colour indicates chlorophyll autofluorescence. Bars = 500 μm in (**a**), 10 μm in (**b**), (**e**), and (**g**–**i**), and 200 nm in (**c**), (**d**), and (**f**).

Distinct from the other MpSYP1 members, MpSYP12B exhibits specific expression in oil body cells and localizes to the oil body membrane (Supplementary Fig. 2b)^17^. Oil body cells expressing a _*pro*_Mp*SYP12B:*2×YFP construct indicated that MpSYP12B distributed around meristematic regions in young thalli, which was observed using light-sheet microscopy (Fig. 2a; Supplementary Movie 1). These results suggest that oil body formation occurs around meristematic regions and into thalli during the growth. mCitrine-MpSYP12B expressed under its own regulatory elements localized to the oil body membrane with a faint signal on the PM (Fig. 2b), which was also confirmed by immune-electron microscopy using an anti-GFP antibody (Fig. 2c, d). We then tested whether organelle markers for the endoplasmic reticulum (MpSEC20, MpUSE1A, MpSEC22, mRFP-HDEL, and GFP-HDEL), Golgi apparatus (MpGOS11 and MpSFT1), *trans*-Golgi network (MpSYP6A and MpSYP4), and tonoplast (MpSYP2 and MpVAMP71) were targeted to the oil body membrane, none of which were detected (Fig. 2e and Supplementary Fig. 2c, d). Using transmission electron microscopy, we found that clathrin-coated vesicles formed from the oil body membrane, and emergence and disappearance of clathrin-positive foci at the oil body membrane was also observed in transgenic plants expressing Citrine-tagged MpCLC1 (Fig. 2g; Supplementary Movie 2).

**Fig. 3:**
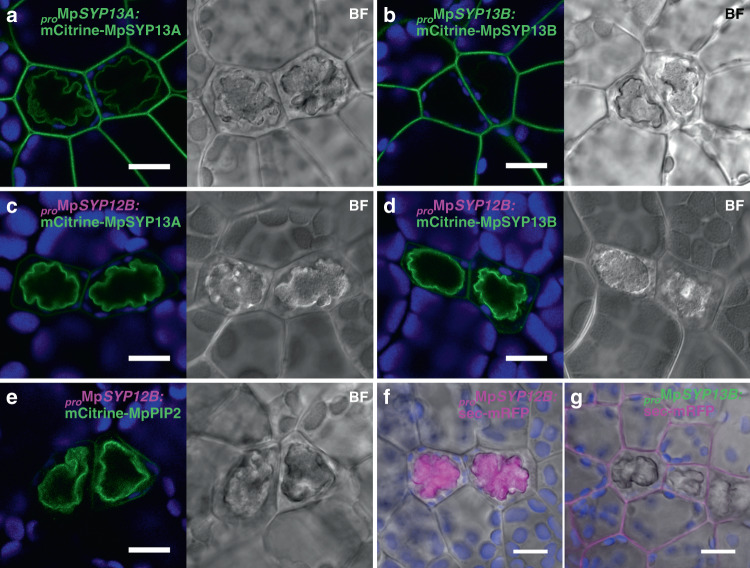
The oil body is formed by redirection of the secretory pathway. **a, b** Thallus cells including oil body cells expressing mCitrine-MpSYP13A (**a**) or mCitrine-MpSYP13B (**b**) under their own regulatory elements. **c**–**e** Thallus cells including oil body cells expressing mCitrine-MpSYP13A (**c**), mCitrine-MpSYP13B (**d**), or mCitrine-MpPIP2 (**e**) under the Mp*SYP12B* promoter. BF, bright-field images. **f**, **g** Thallus cells including oil body cells expressing sec-mRFP under the Mp*SYP12B* promoter (**f**) or the Mp*SYP13B* promoter (**g**). Bright-field images are merged. Bars = 10 μm.

Given that clathrin-mediated endocytosis occurs at the PM, and SYP1 members generally function on the PM, the oil body membrane could share similar characteristics with the PM. This hypothesis was supported by dual localization of the PM proteins, PM-type aquaporin MpPIP2 (Fig. 2h) and PM-resident SNARE MpSYP13A (Fig. 3a) driven by their own regulatory elements. The plasma membrane-like nature of the oil body membrane and the existence of the SYP1 member that is homologous to KNOLLE suggested that the oil body could be formed by the fusion of secretory vesicles similar to the cell plate. Thus, the luminal space of the oil body should be topologically equivalent to the extracellular space, which was tested by expressing a general secretion marker sec-mRFP, which is composed of the signal peptide for ER translocation and mRFP under the constitutive Mp*EF1α* promoter. In non-oil body cells, mRFP fluorescence was detected only in the extracellular space (Supplementary Fig. 2e). However, mRFP accumulated in the oil body in addition to the extracellular space in oil body cells, demonstrating equivalent topology between the lumen of the oil body and extracellular space (Fig. 2i). Unlike MpSYP12A and KNOLLE, MpSYP12B loss of function did not result in a detectable abnormality in oil body formation and transport of MpSYP13A or sec-mRFP (Supplementary Fig. 3), probably reflecting a functional redundancy between MpSYP12B and 13A, similar to the partial functional redundancy of KNOLLE and SYP132^32^.

### The oil body is formed by redirecting the secretory pathway

**Fig. 4:**
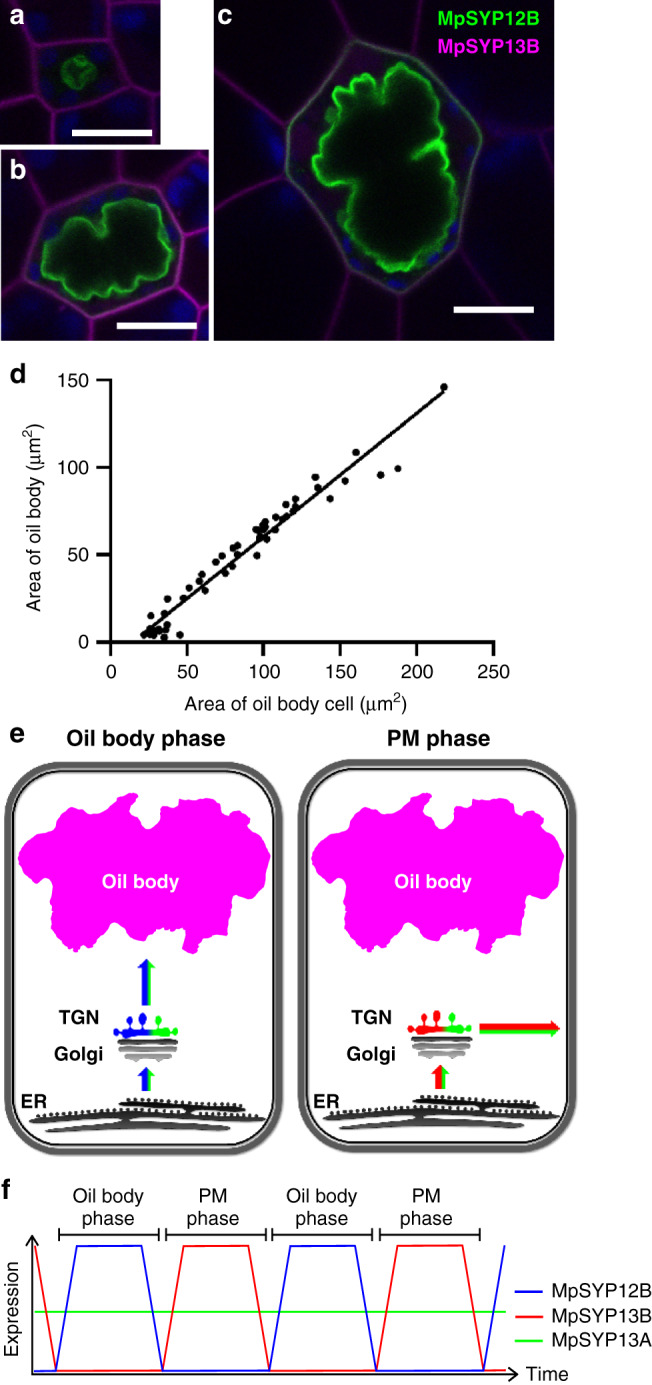
The oil body cycle hypothesis. **a**–**c** Oil body cells at distinct developmental stages expressing mCitrine-MpSYP12B and mRFP-MpSYP13B. Green, magenta, and blue pseudo-colours indicate mCitrine, mRFP, and autofluorescence from chlorophyll, respectively. Bars = 10 μm. **d** A scatter diagram of the areas of oil body cells on the *X* axis and the areas of oil bodies on the *Y* axis. *R*^2^ = 0.9547. **e**, **f** Schemes of the oil body cycle hypothesis. Newly synthesized PM proteins and secreted cargos are targeted to the oil body membrane in the oil body phase, and then to the PM and extracellular space during the PM phase (**e**). Expression levels of MpSYP12B and MpSYP13B periodically oscillate depending on the cell phases; whereas, MpSYP13A is constitutively expressed, resulting in dual localization at the oil body membrane and the PM (**f**).

Distinct from MpSYP13A with the dual localization to the plasma and oil body membranes, the close homolog MpSYP13B was only localized to the PM in oil body cells (Fig. 3a, b). Surprisingly, both of these proteins were targeted predominantly to the oil body membrane when expressed under the regulation of the Mp*SYP12B* promoter (Fig. 3c, d). This effect was not restricted to SYP1 homologs; the PM-resident protein MpPIP2 and a general secretion cargo sec-mRFP were also targeted almost exclusively to the oil body when driven by the Mp*SYP12B* promoter (Fig. 3e, f). These results indicated that the secretory pathway is redirected inward and vesicles fuse with each other to form the oil body during the phase in which the Mp*SYP12B* promoter is active. In contrast, when the Mp*SYP13B* promoter is active, the secretory pathway is directed to the PM and extracellular space, which was further confirmed by the accumulation of sec-mRFP predominantly in the extracellular space when the construct was driven by the Mp*SYP13B* promoter (Fig. 3g). Other organelle markers did not change their localization even when expressed under the Mp*SYP12B* promoter (Supplementary Fig. 2c, d). These results indicate that the redirections of the secretory pathway in oil body cells is regulated at the transcription level. The reorientation of secretory directions should take place periodically and repeatedly through oil body cell development, because we never observed oil body membrane localization for MpSYP13B at any developmental stages of oil body cells (Fig. 4a–c), and increase in the size of the oil body cell along with the increase in the oil body size was observed (Fig. 4d). Based on these findings, we propose the “oil body cycle hypothesis”, which states that Marchantia oil body cells cycle between two distinct cellular phases: the “PM phase” in which the secretory pathway is oriented to the PM and extracellular space, and the “oil body phase” when the secretory pathway is oriented to the oil body, with the phase transition under the regulation of a transcriptional regulatory system (Fig. 4e, f).

### MpERF13 transcription factor regulates oil body formation

**Fig. 5:**
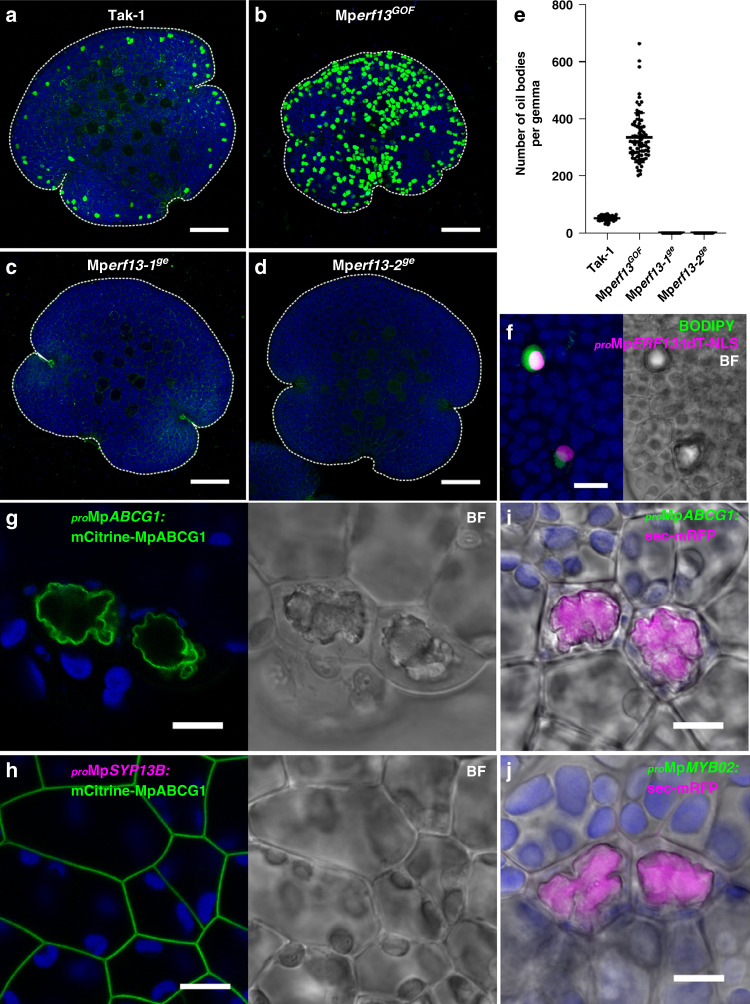
MpERF13 transcription factor regulates oil body formation. **a**–**d** Maximum intensity projection images of BODIPY-stained gemmae of Tak-1 (**a**), Mp*erf13*^*GOF*^ (**b**), Mp*erf13-1*^*ge*^ (**c**), and Mp*erf13-2*^*ge*^ (**d**). **e** The number of oil bodies visualized by BODIPY-staining in gemmae. Bars indicate means ± s.d. Statistical analyses between Tak-1 and each genotype were conducted using a two-tailed Welch’s *t*-test. Sample numbers were 57 gemmae for Tak-1, 78 for Mp*erf13*^*GOF*^, 61 for Mp*erf13-1*^*ge*^, and 75 for Mp*erf13-2*^*ge*^; *p*-values are 1.19 × 10^−43^ for Mp*erf13*^*GOF*^, 1.44 × 10^−45^ for Mp*erf13-1*^*ge*^, and 1.44 × 10^−45^ for Mp*erf13-2*^*ge*^. **f** A maximum intensity projection image of oil body and non-oil body cells in a gemma expressing tandem Tomato (tdT)-NLS driven by the Mp*ERF13* promoter. **g**, **h** Thallus cells including oil body cells expressing mCitrine-MpABCG1 under its own regulatory elements (**g**) and the Mp*SYP13B* promoter (**h**). BF, bright-field images. **i**, **j** Thallus cells expressing sec-mRFP under the Mp*ABCG1* (**i**) and the Mp*MYB02* (**j**) promoters. Bright-field images are merged. Green, magenta, and blue pseudo-colours indicate fluorescence from BODIPY or mCitrine, tdT or mRFP, and chlorophyll, respectively. Bars = 100 μm in (**a**– **d**) and 10 μm in (**f**–**j**).

To investigate the regulatory system of oil body formation and the oil body cycle, we screened mutants defective in oil body formation (Supplementary Fig. 4a). From 48,825 T-DNA insertion lines, we identified a mutant (Mp*erf13*^*GOF*^) with an increased number of oil bodies. The mutant gemmae contained 334.4 ± 85.6 oil bodies (mean ± s.d.), whereas wild-type gemmae possessed 51.9 ± 8.7 oil bodies under our experimental conditions (Fig. 5a–e). The T-DNA was inserted 2674-bp upstream of the start codon of Mapoly0060s0052.1 (Mp*ERF13*), which encodes a putative ERF/AP2 transcription factor in the subgroup containing DREB1A and TINY in Arabidopsis (Supplementary Fig. 4b). Moreover, Mp*ERF13* and Mp*SYP12B* transcripts accumulated in this mutant, compared to the wild type, as detected by RNA-sequencing (RNA-Seq) and quantitative RT-PCR analyses (Supplementary Fig. 5b). We then generated knockout mutants in which the Mp*ERF13* gene was deleted by genome editing (Supplementary Fig. 4c). Two independent mutants (Mp*erf13-1*^*ge*^ and Mp*erf13-2*^*ge*^) exhibited no detectable abnormalities in thallus development and reproductive growth; however, these mutants completely lacked oil bodies in the gemma and thalli (Fig. 5c–e and Supplementary Fig. 4f, g). These phenotypes of gain- and loss-of-function mutations suggested that MpERF13 is a major transcription factor regulating oil body formation. Consistently, the Mp*ERF13* promoter was highly active in oil body cells (Fig. 5f).

As MpERF13 is homologous to ERF/AP2 transcription factors, this protein was expected to be involved in the transcriptional regulation of oil body formation and/or the oil body cycle. To identify genes downstream of MpERF13, we analysed transcriptomes in Tak-1 (wild type) and Mp*erf13*^*GOF*^ and Mp*erf13-1*^*ge*^ mutant lines by RNA-Seq. Through the comparison between the three groups using an ANOVA-like test, we identified 136 differentially expressed genes (DEGs) other than Mp*ERF13* whose expression was higher than two log_2_-fold change (FC) in Mp*erf13*^*GOF*^ compared with Tak-1, and lower than -2 log_2_FC in Mp*erf13-1*^*ge*^ than Tak-1 (FDR < 0.01) (Supplementary Data 1). To verify the RNA-Seq result, we also performed the quantitative RT-PCR analysis for selected 11 DEGs including Mp*ERF13* and Mp*SYP12B*, which confirmed that all of these DEGs were highly expressed in Mp*erf13*^*GOF*^, but their expression was significantly lower or not detectable in Mp*erf13-1*^*ge*^ and Mp*erf13-2*^*ge*^ compared with Tak-1 (Supplementary Fig. 5). These data strongly suggested that these 136 genes function downstream of Mp*ERF13* in oil body cells, which was further verified for the two genes Mapoly0083s0014.1 and MpMYB02 (Mapoly0006s0226). Mapoly0083s0014.1 was positively regulated by MpERF13 and encodes a member of the G subfamily of ABC transporters^27^, which we named MpABCG1. Although ABCG members are generally targeted to the PM and mediate efflux of secondary metabolites from intracellular to extracellular spaces^33,34^, mCitrine-MpABCG1 was targeted to the oil body membrane when expressed under its own regulatory elements (Fig. 5g); however, this protein was targeted to the PM in non-oil body cells when expressed under the Mp*SYP13B* promoter (Fig. 5h). MpMYB02, whose expression was also positively regulated by MpERF13, is reported to be a transcription factor responsible for the production of marchantin A and its derivatives that accumulate in oil bodies^15,35^. Mp*ABCG1* and Mp*MYB02* are specifically expressed in oil body cells and when sec-mRFP was driven by the promoters of these genes, it was specifically targeted to the lumen of oil bodies. These results indicate that MpABCG1 and MpMYB02, presumably together with other DEGs we identified, act during oil body formation downstream of MpERF13.

**Fig. 6:**
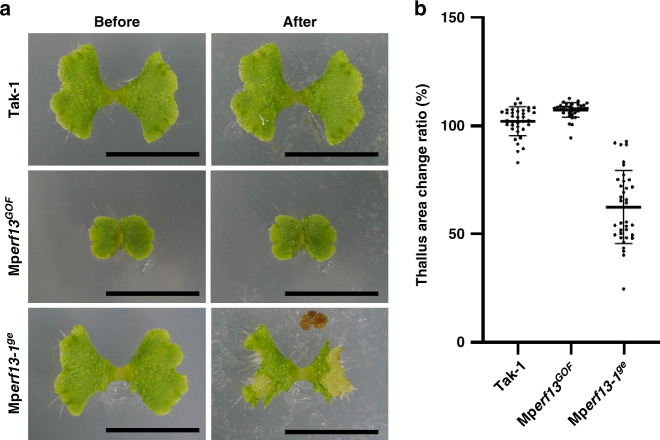
Loss of the oil body increases Marchantia herbivory. **a** Ten-day-old thalli in indicated genotypes before they were fed to starved pill bugs (Before) or 24 h after feeding (After). **b** Ratios of thallus areas of After: Before pill bug feeding were calculated to indicate thallus area change ratios (*n* = 36 thalli for each genotype). Bars indicate means ± s.d. Statistical analyses between Tak-1 and each genotype were conducted by two-tailed Welch’s *t*-test. The *p*-values were 1.07 × 10^−4^ for Mp*erf13*^*GOF*^ and 4.10 × 10^−17^ for Mp*erf13-1*^*ge*^. Bars = 1 cm.

Lastly, we asked why liverworts possess oil bodies. Using fresh and alcohol-washed liverworts, the oil body was proposed to be an effective chemical protection from herbivores^36^, which was also recently supported by a feeding assay using *Armadillidium vulgare* (pill bug)^37^. We fed starved pill bugs with wild-type liverwort and genetically established mutants that contain extra or no oil bodies. Thalli of Tak-1 and Mp*erf13*^*GOF*^ remained almost intact after 24 h of herbivory, while the areas of Mp*erf13-1*^*ge*^ and Mp*erf13-2*^*ge*^ thalli were significantly reduced after herbivory (Fig. 6 and Supplementary Fig. 6). This result further supports that the oil body is effective in protecting liverworts from herbivores.

## Discussion

Plant SYP1 family generally localizes to the PM and execute membrane fusion between the PM and secretory vesicles. The SYP1 family, which has expanded during land plant evolution, consists of two subgroups, the SYP13 and SYP11/12 groups. The SYP11/12 group in seed plants is further divided into two subclades with distinct functions, SYP11 and SYP12 groups, suggesting sub- and/or neofunctionalization in the SYP11/12 group in an ancestor of seed plants (Supplementary Fig. 1a)^17,26^. Another key machinery component of membrane trafficking, RAB GTPase, is also shown to have been expanded during land plant evolution, in which secretory RAB GTPases including RABA/RAB11 exhibit remarkable functional diversification^38,39^. Cell plate formation during cytokinesis involves distinctive subgroups of the SYP1 and RAB11 families^28,40,41^, probably reflecting that diversified SYP1 and RAB11 members were co-opted to the trafficking pathway to the cell plate. In this study, we found that an organelle specific to liverworts, the oil body, should also be acquired through paralogous expansion followed by neofunctionalization of the SYP1 group. Although the functions of the cell plate and oil body are totally different, they share several important traits that include specific SYP11/12 members that reside at their membranes, clathrin-coated vesicles generated from the plasma and oil body membranes, and both the cell plate and oil body are formed by reorientation of secretory pathways. In both cases, the orientation of the secretory pathway is redirected in response to transitioning cellular states during the cell cycle for cell plate formation and the hypothetical oil body cycle for oil body formation. The orientation of the plant secretory pathway is also modulated during plant-microbe interactions; symbiotic arbuscular mycorrhizal fungi redirects the plant secretory pathway to form the arbuscule in host plant cells that acts as an interface for sugar and mineral exchange^42,43^. Thus, the redirection of the secretory pathway is a common strategy for plants to acquire novel organelles/cellular structures that have resulted in maximizing fitness for land plants during evolution. A lineage-specific acquisition of secretion-related organelles associated with duplication and neofunctionalization of trafficking machineries, such as RAB GTPase, was also reported in protozoa^6,44^. Thus, acquisition of new organelles by redirection of the secretory pathway through the paralogous expansion of trafficking machinery components followed by their neofunctionalization would be a commonly adopted evolutionary path, concordant with the organelle paralogy hypothesis.

The oil bodies in liverworts contain a variety of secondary metabolites such as sesquiterpenoids and bisbibenzyls^11,15^, whose biological functions remain to be determined. In this study, we demonstrated that the oil body reduces Marchantia herbivory using mutant plants with excess or without oil bodies. Consistently, a mutant of the MpC1HDZ transcription factor with a reduced number of the oil body was also recently shown to exhibit increased herbivory^37^. The secondary metabolites stored in the liverwort oil body were also reported to exhibit a diverse range of bioactivities, e.g., anti-cancer and anti-virus activities^11^. Thus, modulation of the oil body formation using MpERF13 would provide a promising methodology for the pharmaceutical application of these compounds. We also demonstrated in this study that a general secretion marker sec-mRFP was targeted to the luminal space of the oil body when expressed during the hypothetical oil body phase, suggesting that any soluble proteins could be targeted and stored in the oil body just by adding the signal peptide for translocation to the ER lumen. The knowledge obtained from the study of the Marchantia oil body, in combination with attempts to modulate organelle biogenesis using other tools^45^, would open a new road to engineering organelle functions to maximize and/or design plant cellular functions.

## Methods

### Phylogenetic analyses

Phylogenetic analyses were performed as previously described in Bowman et al.^27^ with minor modification. Previously published datasets in Kanazawa et al.^17^ and Bowman et al.^27^, were used for phylogenetic analyses of SYP1 and ERF/AP2 members, respectively. Multiple sequence alignments were performed using the MUSCLE program version 3.8.31^46,47^ with the default parameter. The alignment gaps were removed by Gblocks program version 0.91b^48,49^ or manually. The maximum likelihood phylogenetic analyses were performed using PhyML 3.0^50^ under the LG model. Bootstrap analyses were performed by resampling 1000 sets.

### Vector construction

Open reading frames (ORFs) and genomic sequences of Marchantia genes were amplified by polymerase chain reaction (PCR) from cDNA and genomic DNA prepared from the accession Tak-1; the amplified products were subcloned into pENTR/D-TOPO (ThermoFisher, Waltham, USA) according to the manufacturer’s instructions. The primer sequences and sizes of amplified products are listed in Supplementary Data 2. For the construction of pENTR _*pro*_Mp*ERF13*, the 5′ sequence (promoter + 5′ UTR) was amplified and subcloned into pENTR/D-TOPO. The resultant sequence was introduced into pMpGWB316^51^ using the Gateway LR Clonase™ II Enzyme Mix (ThermoFisher) according to the manufacturer’s instructions. For the construction of pENTR genomic mGFP-MpSYP12A and mRFP-MpSYP13B constructs, the cDNA for mGFP and mRFP were inserted into *Sma*I and *Bam*HI sites in the pENTR genomic XFP(*Sma*I)-MpSYP12A and pENTR genomic XFP(*Bam*HI)-MpSYP13B, which were previously prepared^17^ using the In-Fusion HD Cloning System (Clontech, Shiga, Japan). For the construction of pENTR genomic mCitrine-MpPIP2, pENTR genomic mCitrine-MpSYP4, pENTR genomic mCitrine-MpSEC22, and pENTR genomic mCitrine-MpABCG1, genomic sequences of the protein-coding regions and 3′ flanking sequences were amplified with a *Sma*I site at the 5′ end and subcloned into the pENTR vector. The 5′ sequences (promoter + 5′ UTR) and cDNA of mCitrine were amplified and inserted into *Not*I and *Sma*I sites, respectively, of the resultant pENTR vectors using the In-Fusion HD Cloning System. The resultant chimeric genes were then introduced into pMpGWB301^51^ using the Gateway LR Clonase™ II Enzyme Mix. For the construction of pENTR _*pro*_Mp*SYP12B:mCitrine*, the _*pro*_Mp*SYP12B:mCitrine* sequence was amplified from the pENTR genomic mCitrine-MpSYP12B vector, and subcloned into pENTR/D-TOPO. The resultant sequence was introduced into pMpGWB307^51^ using the Gateway LR Clonase™ II Enzyme Mix to create the pMpGWB307 _*pro*_Mp*SYP12B:2*×*YFP* construct. For the construction of pENTR genomic MpCLC1-mCitrine, the genomic sequence comprising the 5′ flanking (promoter + 5′ UTR) and protein-coding sequences were amplified with a *Sma*I site at the 3′ end and subcloned into the pENTR vector. The 3′ flanking sequence including 3′ UTR and cDNA for mCitrine were amplified and inserted into the *Asc*I and *Sma*I sites, respectively, of the resultant pENTR vectors using the In-Fusion HD Cloning System. The resultant chimeric genes were then introduced into pMpGWB301^51^ using the Gateway LR Clonase™ II Enzyme Mix. For the construction of pENTR sec-mRFP, sequences for the signal peptide and mRFP were independently amplified by PCR, and the sequence of sec-mRFP was then amplified by PCR using the mixture of the amplified products as templates. The amplified products were subcloned into pENTR/D-TOPO. For the construction of pENTR mRFP-HDEL, the sequence of SP-mRFP-HDEL was amplified by PCR using pENTR sec-mRFP as template, and subcloned into pENTR/D-TOPO. For the construction of pENTR _*pro*_Mp*SYP12B:*sec-mRFP, pENTR _*pro*_Mp*SYP13B:*sec-mRFP, pENTR _*pro*_Mp*ABCG1:*sec-mRFP, and pENTR _*pro*_Mp*MYB02:*sec-mRFP, the 5′ sequences (promoter + 5′ UTR) were amplified and were inserted into *Not*I sites of the pENTR sec-mRFP vector using the In-Fusion HD Cloning System. The resultant sequences were then introduced into pMpGWB301^51^ using the Gateway LR Clonase™ II Enzyme Mix. To construct pMpGWB301-derived Gateway vectors (Supplementary Fig. 2a), the promoter*:mCitrine* sequences were amplified from the genomic constructs described above, and inserted at a *Hin*dIII site of pMpGWB301 using the In-Fusion HD Cloning System. pENTR MpTUB2^52,53^ was kindly provided from Dr. R. Nishihama and introduced into pMpGWB301_*pro*_Mp*SYP2:mCitrine*-GW (Supplementary Fig. 2a) using the Gateway LR Clonase™ II Enzyme Mix. For construction of the genome-editing vectors, the target sequences were selected using CRISPR direct (https://crispr.dbcls.jp/)^54^; double-stranded oligonucleotides of the target sequences were inserted into the pMpGE_En03 vector^55^. The resultant gRNA cassettes were introduced into the pMpGE010 or pMpGE011 vectors^55^ using the Gateway LR Clonase II Enzyme Mix. For the construction of the homologous recombination-mediated gene targeting vector, the 3.5-kb homologous genomic sequences were amplified from the Tak-1 genome and inserted at *Pac*I and *Asc*I sites of the pJHY-TMp1 vector^56^ using the In-Fusion HD Cloning System.

### Plant materials and transformation

Marchantia accession Takaragaike-1 (Tak-1, male) and Takaragaike-2 (Tak-2, female)^57^ were used throughout the study. The growth conditions and transformation methods were previously described^17,57,58^. Thalli were grown asexually and maintained on 1/2× Gamborg’s B5 medium containing 1.0% (w/v) agar at 22 °C under continuous white light. The transition from the vegetative to reproductive growth was induced by supplementing far-red light, and spores were generated by crossing male and female lines. For transformation using regenerating thalli, 11-day-old thalli, from which apical regions including meristems were removed, were cultured on 1/2× Gamborg’s B5 medium containing 1.0% (w/v) sucrose and 1.0% (w/v) agar at 22 °C for three days under continuous white light for regeneration. The regenerating plantlets were co-cultured with the agrobacterium strain GV2260 harbouring a binary vector in the liquid 0M51C medium containing 2% (w/v) sucrose and 100 μM acetosyringone at 22 °C under continuous white light with agitation at 130 rpm for three days. Transformants were selected on 1/2× Gamborg’s B5 medium plates containing 10 mg L^−1^ hygromycin B and 100 mg L^−1^ cefotaxime for pMpGWB101, pMpGWB103, pMpGE010, and pJHY-TMp1 vectors, and on the medium plates plus 0.5 μM chlorsulfuron and 100 mg L^−1^ cefotaxime for pMpGWB301, pMpGWB307, pMpGWB316, and pMpGE011, respectively. The method of transformation using sporelings is described in the forward genetic screening for the oil body formation mutants section. *M*. *polymorpha* expressing mCitrine-MpSYP12A, mCitrine-MpSYP12B, mCitrine-MpSYP13A, mCitrine-MpSYP13B, mCitrine-MpSYP2 or mCitrine-MpVAMP71 under the regulation of their own regulatory elements (e.g. promoter, UTRs, and introns), and *M*. *polymorpha* expressing mRFP-MpSYP6A or GFP-HDEL driven by the Mp*EF1α* promoter were previously reported^17,51,59^.

### Genotyping

Genomic DNA was extracted from the thalli in an extraction buffer containing 1 M KCl, 100 mM Tris-HCl (pH 9.5), and 10 mM EDTA. This DNA was used as templates for PCR. Genome fragments with putative mutation sites were amplified by PCR using KOD FX Neo (TOYOBO, Osaka, Japan) according to the manufacturer’s instructions. The primers used in PCR-based genotyping are listed in Supplementary Data 2.

### Confocal laser scanning microscopy

Two- and five-day-old thalli were used for imaging the cell plate and the oil body, respectively. Samples were mounted in distilled water and observed using an LSM780 confocal microscope (Carl Zeiss) equipped with an oil immersion lens (×63, numerical aperture = 1.4) on an electrically driven stage. Gemmae were observed with a ×10 objective lens (numerical aperture  = 0.45). Thalli were incubated in 10 μM *N*-(3-triethylammoniumpropyl)-4-(6-(4-(diethylamino)phenyl)hexatrienyl) pyridinium dibromide (FM4-64, Thermo Fisher) solution for 2 min. Samples were then washed twice in water before observation. For 4,4-difluoro-1,3,5,7,8-pentamethyl-4-bora-3a,4a-diaza-*s*-indacene (BODIPY 493/503, Thermo Fisher) staining, thalli or gemmae were incubated in 200 nM BODIPY 493/503 dissolved in water for 10 min and then washed twice in water before observation. The samples were excited at 488 nm (Argon 488) and 561 nm (DPSS 561-10) lasers, respectively, and the fluorescent emission was collected between 482-659 nm using twenty GaAsP detectors. Spectral unmixing and the construction of maximum intensity projection images were conducted using the ZEN2012 software (Carl Zeiss). Image processing was performed with ZEN2012 software, Photoshop software (Adobe Systems), and ImageJ (National Institute of Health, https://imagej.nih.gov/ij/).

### Electron microscopy

For immunoelectron microscopy, five-day-old *M*. *polymorpha* thalli from Tak-1 and thalli expressing mCitrine-MpSYP12B were used. The sandwiched samples with copper disks were frozen in liquid propane at −175 °C. After the samples were frozen, they were freeze-substituted with 0.5% tannic acid in acetone and 3% distilled water at −80 °C for 48 h. The samples were then kept at −20 °C for 3 h followed by incubation at 4 °C for 1 h. Next, the samples were dehydrated in anhydrous ethanol 3 times for 1 h each, followed by infiltration with a 50:50 mixture of ethanol and LR white resin at 4 °C for 3 h. The samples were transferred to new 100% LR white resin and incubated at 4 °C for 1 h, and this process was repeated three times. Next, the samples were transferred to fresh 100% resin and polymerized at 50 °C overnight. The blocks were sliced into ultra-thin (80-nm) sections using an ultramicrotome, and the sections were placed on nickel grids. The grids were then incubated with the primary antibody (Anti-GFP polyclonal antibody, ab6556, abcam) in PBS plus 1% BSA for 90 min at room temperature, followed by three rinses with PBS plus 1% BSA for 1 min each. The grids were then floated on drops of secondary antibody conjugated with gold particles (10 nm) (anti-rabbit IgG polyclonal antibody, EM.GAR10, BBI Solutions) for 1 h at room temperature. The primary and secondary antibodies were used at 1:50 and 1:20, respectively. After rinsing with PBS, the grids were placed in 2% glutaraldehyde in 0.1 M phosphate buffer, dried, stained with 2% uranyl acetate for 15 min, rinsed with distilled water, and then subjected to secondary staining with lead stain solution at room temperature for 3 min. The grids were observed using a transmission electron microscope (JEM-1200EX; JEOL Ltd.) at an acceleration voltage of 80 kV. Digital images were obtained with a CCD camera (VELETA, Olympus Soft Imaging Solutions GmbH).

For morphological observation of the oil body, five-day-old Tak-1 thalli were fixed with 2% paraformaldehyde and 2% glutaraldehyde in 0.05 M cacodylate buffer pH 7.4 at 4 °C overnight. The fixed samples were washed three times with 0.05 M cacodylate buffer for 30 min each and were then post-fixed with 2% osmium tetroxide in 0.05 M cacodylate buffer at 4 °C for 3 h. The samples were dehydrated in graded ethanol solutions (50% and 70% ethanol for 30 min each at 4 °C, 90% for 30 min at room temperature, four times with 100% for 30 min each at room temperature, and 100% overnight at room temperature). The samples were infiltrated with propylene oxide (PO) two times for 30 min each, and then placed into a 70:30 mixture of PO and resin (Quetol-651; Nisshin EM Co.) for 1 h. The caps of tubes were opened overnight to volatilize PO. The samples were transferred to fresh 100% resin and polymerized at 60 °C for 48 h. Ultra-thin sample sections were mounted on copper grids, stained with 2% uranyl acetate and lead stain solution (Sigma-Aldrich), and observed under a transmission electron microscope (JEM-1400Plus; JEOL Ltd) at an acceleration voltage of 80 kV. Digital images (2048 × 2048 pixels) were obtained using a CCD camera (VELETA; Olympus Soft Imaging Solutions GmbH).

### Light-sheet microscopy

Five-day-old thalli expressing 2×Citrine driven by the Mp*SYP12B* promoter were embedded in low-melt agarose gel and observed using a Light-sheet Z.1 microscope (Carl Zeiss) equipped with a water immersion lens (×5, numerical aperture = 0.16). The samples were excited at 488 nm (Argon 488 laser). Acquisition and construction of three-dimension images from multi-angle images were conducted using the ZEN2014 software (Carl Zeiss). The images were processed digitally with Imaris 8.2 (Bitplane) and Photoshop software.

### Fluorescent stereoscopic microscopy

Three-week-old wild-type and Mp*erf13*^*ge*^ thalli were stained by BODIPY493/503 (ThermoFisher) and observed using a fluorescent stereoscopic microscope (M165 FC, Leica) equipped with a MC170 HD digital camera (Leica).

### Forward genetic screening for the oil body formation mutants

The workflow of the mutant screening is described in Supplementary Fig. 4a. Liverworts with transfer DNA (T-DNA) insertions were generated by co-culture of *M*. *polymorpha* sporelings with the agrobacteria strain GV2260 harbouring the binary vector pCAMBIA1300 (https://cambia.org/welcome-to-cambialabs/cambialabs-projects/cambialabs-projects-legacy-pcambia-vectors-pcambia-legacy-vectors-1/cambialabs-projects-legacy-pcambia-vectors-list-of-legacy-pcambia-vectors-3/). T_1_ plants were first selected on 1/2× Gamborg’s B5 plates containing 10 mg L^−1^ hygromycin and 100 mg L^−1^ cefotaxime. Hygromycin-resistant T_1_ plants were then transferred and incubated on hygromycin-free medium for about one week, followed by BODIPY 493/503 staining to visualize oil bodies. Visible screening for mutants defective in oil body formation was performed using a fluorescent stereoscopic microscope (M165 FC, Leica). To identify flanking sequences of T-DNA insertions, thermal asymmetric interlaced-PCR (TAIL-PCR) was performed as described previously^57,60,61^ with minor modifications using crude-extracted DNA as templates. Flanking sequences were amplified using KOD FX neo DNA polymerase and T-DNA-specific (TR1–3 and TL1–3 for right and left borders of T-DNA, respectively) and universal adaptor (AD1–6) primers. The reaction cycles are shown in Supplementary Data 3. After agarose gel electrophoresis of the final TAIL-PCR products, DNA bands were excised and purified using the Wizard SV Gel and PCR Clean-Up System (Promega). Purified products were directly sequenced using TR3 or TL3 primers. The T-DNA insertion sites were identified using the genome sequence registered in MarpolBase, genome version 3.1.

### RNA extraction and RNA-sequencing analysis

Total RNA from Tak-1, Mp*erf13*^*GOF*^ and Mp*erf13-1*^*ge*^ thalli was extracted by RNeasy (QIAGEN) according to the manufacturer’s instructions. The eluted total RNA samples were treated with DNase I (Takara) to remove DNA contamination. The quality and quantity of the total RNA were evaluated with a NanoDrop 1000 spectrophotometer (Thermo Fisher Scientific), Qubit 2.0 Fluorometer (Thermo Fisher Scientific) and a Bioanalyzer RNA6000 Nano Kit (Agilent Technologies). The sequence libraries were prepared with the TruSeq RNA Sample Prep Kit v2 (Illumina) according to the manufacturer’s low sample protocol. The quality and quantity of each library were determined using a Bioanalyzer with the High Sensitivity DNA kit (Agilent Technologies) and the KAPA Library Quantification Kit for Illumina (Illumina). Equal amounts of each library were mixed to make the 2 nM pooled library. Illumina sequencing was performed using a HiSeq 1500 platform (Illumina) to produce 126-bp single-end reads. Three biological replicates were prepared for the library construction and RNA-Seq analysis. All reads are available through the Sequence Read Archive (SRA) under the accession number DRA009193. The abundance of transcripts from Illumina RNA-Seq data were quantified with the kallisto program (version 0.43.1)^62^ with the default parameters using the primary transcript dataset from the Marchantia genome version 3.1. To estimate DEGs, an ANOVA-like test was performed by edgeR (version 3.18.1)^63^. Genes with FDR values lower than 0.01 and an absolute log_2_ FC > 2 were considered to be differentially expressed. Among all combinations of DEGs (log_2_FC(Mp*erf13*^*GOF*^/Tak-1) > 2 and log_2_FC(Mp*erf13-1*^*ge*^/Tak-1) > 2, log_2_FC(Mp*erf13*^*GOF*^/Tak-1) > 2 and log_2_FC(Mp*erf13-1*^*ge*^/Tak-1) < -2, log_2_FC(Mp*erf13*^*GOF*^/Tak-1) < −2 and log_2_FC(Mp*erf13-1*^*ge*^/Tak-1) > 2, log_2_FC(Mp*erf13*^*GOF*^/Tak-1) < −2 and log_2_FC(Mp*erf13-1*^*ge*^/Tak-1) < −2), we selected the set log_2_FC(Mp*erf13*^*GOF*^/Tak-1) > 2 and log_2_FC(Mp*erf13-1*^*ge*^/Tak-1) < −2 as candidate genes regulated downstream of MpERF13, which included both Mp*ERF13* and Mp*SYP12B*.

### Quantitative reverse transcription (qRT)-PCR

For qRT-PCR, cDNA was synthesized from total RNA using SuperScript III Reverse Transcriptase (Invitrogen) and an oligo dT(18) primer according to the manufacturer’s instructions. qRT-PCR was performed with LightCycler 480 (Roche) using FastStart SYBR Green Master (Roche) according to the manufacturer’s protocol. Sequences of primers used are listed in Supplementary Data 2. Mp*APT* (Mapoly0100s0027.1/Mp3g25140.1) was used as a housekeeping reference for normalization^64^. Three biological replicates were prepared, and three technical replicates were performed for each reaction.

### Pill bug feeding assay

The feeding assay was performed according to Nakazaki et al.^65^ with modification. Gemmae were cultured on 1/2× Gamborg’s B5 medium containing 1% (w/v) agar and 1% (w/v) sucrose for five days at 22 °C under continuous white light (50 μmol m^−2 ^s^−1^). Five-day-old thalli were transferred onto 1/2× Gamborg’s B5 medium containing 1% (w/v) agar without sucrose and cultured for additional five days under the same conditions. Pill bugs (*Armadillidium vulgare*) were collected in the Myodaiji area of NIBB (Nishigonaka 38, Myodaiji, Okazaki 444-8585 Aichi, Japan). Pill bugs were maintained on Prowipe (Daio Paper, Tokyo, Japan) moistened with sterilized water for 48 h at 22 °C in the dark without feeding before the assay. Six pill bugs were introduced into each medium plate containing 10-day-old thalli and kept for 24 h in the dark at 22 °C. The thallus areas were calculated using ImageJ (National Institute of Health, https://imagej.nih.gov/ij/).

### Gene accession numbers

Accession numbers of genes used in this study are listed in Supplementary Data 4. We followed the nomenclature of genes, proteins, and mutants of Marchantia reported in Bowman et al.^66^. Gene IDs were taken from MarpolBase (http://marchantia.info/), genome version 3.1 and version 5.1^27,67^.

### Statistics and reproducibility

For confocal and electron microscopic images, we presented representative images among biological and technical replicates. The numbers of generated transgenic lines and observed cells are listed in Supplementary Data 5. Statistics were calculated with Excel 2016, and graphs were drawn using Excel 2016 or GraphPad Prism 8.4.3.

### Reporting summary

Further information on research design is available in the Nature Research Reporting Summary linked to this article.

## Supplementary information

Supplementary Information

Descriptions of Additional Supplementary Files

Supplementary Data 1

Supplementary Data 2

Supplementary Data 3

Supplementary Data 4

Supplementary Data 5

Supplementary Movie 1

Supplementary Movie 2

Reporting Summary

## Data Availability

All reads of RNA-Seq are available through the Sequence Read Archive (SRA) under the accession number DRA009193. Source data are provided with this paper.

## Source data

Source Data

## References

[1] Koumandou VL (2013). Molecular paleontology and complexity in the last eukaryotic common ancestor. Crit. Rev. Biochem. Mol. Biol..

[2] Kanazawa T, Ueda T (2017). Exocytic trafficking pathways in plants: why and how they are redirected. N. Phytologist.

[3] Klinger CM, Nisbet RE, Ouologuem DT, Roos DS, Dacks JB (2013). Cryptic organelle homology in apicomplexan parasites: insights from evolutionary cell biology. Curr. Opin. Microbiol..

[4] L’Hernault, S. W. Spermatogenesis. *WormBook*10.1895/wormbook.1.85.1 (2006). 10.1895/wormbook.1.85.1 PMC4781361 18050478

[5] Dacks JB, Field MC (2007). Evolution of the eukaryotic membrane-trafficking system: origin, tempo and mode. J. Cell Sci..

[6] More K, Klinger CM, Barlow LD, Dacks JB (2020). Evolution and natural history of membrane trafficking in eukaryotes. Curr. Biol..

[7] Schlacht A, Herman EK, Klute MJ, Field MC, Dacks JB (2014). Missing pieces of an ancient puzzle: evolution of the eukaryotic membrane-trafficking system. Csh Perspect. Biol..

[8] Shimada T, Takagi J, Ichino T, Shirakawa M, Hara-Nishimura I (2018). Plant vacuoles. Annu. Rev. Plant Biol..

[9] Minamino N, Ueda T (2019). RAB GTPases and their effectors in plant endosomal transport. Curr. Opin. Plant Biol..

[10] Müller S, Jürgens G (2016). Plant cytokinesis-No ring, no constriction but centrifugal construction of the partitioning membrane. Semin. Cell Dev. Biol..

[11] Asakawa Y, Ludwiczuk A, Nagashima F (2013). Phytochemical and biological studies of bryophytes. Phytochemistry.

[12] Galatis B, Apostolakos P, Katsaros C (1978). Ultrastructural Studies on Oil Bodies of Marchantia-Paleacea Bert .1. Early Stages of Oil-Body Cell-Differentiation - Origination of Oil Body. Can. J. Bot..

[13] He XL, Sun Y, Zhu RL (2013). The oil bodies of liverworts: unique and important organelles in land plants. Crit. Rev. Plant Sci..

[14] Suire, C. A comparative, transmission-electron microscopic study on the formation of oil-bodies in liverworts. *J. Hattori. Bot. Lab*. 209–232 (2000).

[15] Tanaka M (2016). Direct evidence of specific localization of sesquiterpenes and marchantin A in oil body cells of Marchantia polymorpha L. Phytochemistry.

[16] Hübener, J. W. P. *Hepaticologia germanica, oder Beschreibung der Deutschen lebermoose*. (Schwan & Götz’sche Hofbuchlandlung, Mannheim, 1834).

[17] Kanazawa T (2016). SNARE molecules in marchantia polymorpha: unique and conserved features of the membrane fusion machinery. Plant Cell Physiol..

[18] Assaad FF (2004). The PEN1 syntaxin defines a novel cellular compartment upon fungal attack and is required for the timely assembly of papillae. Mol. Biol. Cell.

[19] Collins NC (2003). SNARE-protein-mediated disease resistance at the plant cell wall. Nature.

[20] Enami K (2009). Differential expression control and polarized distribution of plasma membrane-resident SYP1 SNAREs in *Arabidopsis thaliana*. Plant cell Physiol..

[21] Grefen C (2015). A vesicle-trafficking protein commandeers Kv channel voltage sensors for voltage-dependent secretion. Nat. Plants.

[22] Lauber MH (1997). The Arabidopsis KNOLLE protein is a cytokinesis-specific syntaxin. J. Cell Biol..

[23] Lukowitz W, Mayer U, Jurgens G (1996). Cytokinesis in the *Arabidopsis embryo* involves the syntaxin-related KNOLLE gene product. Cell.

[24] Ichikawa M (2014). Syntaxin of plant proteins SYP123 and SYP132 mediate root hair tip growth in *Arabidopsis thaliana*. Plant Cell Physiol..

[25] Silva PA, Ul-Rehman R, Rato C, Di Sansebastiano GP, Malho R (2010). Asymmetric localization of Arabidopsis SYP124 syntaxin at the pollen tube apical and sub-apical zones is involved in tip growth. Bmc Plant Biol..

[26] Slane D, Reichardt I, El Kasmi F, Bayer M, Jurgens G (2017). Evolutionarily diverse SYP1 Qa-SNAREs jointly sustain pollen tube growth in *Arabidopsis*. Plant J.: Cell Mol. Biol..

[27] Bowman JL (2017). Insights into land plant evolution garnered from the marchantia polymorpha genome. Cell.

[28] Reichardt I (2011). Mechanisms of functional specificity among plasma-membrane syntaxins in *Arabidopsis*. Traffic.

[29] Ito E (2012). Dynamic behavior of clathrin in Arabidopsis thaliana unveiled by live imaging. Plant J.: Cell Mol. Biol..

[30] Otegui MS, Mastronarde DN, Kang BH, Bednarek SY, Staehelin LA (2001). Three-dimensional analysis of syncytial-type cell plates during endosperm cellularization visualized by high resolution electron tomography. Plant Cell.

[31] Samuels AL, Giddings TH, Staehelin LA (1995). Cytokinesis in tobacco BY-2 and root tip cells: a new model of cell plate formation in higher plants. J. Cell Biol..

[32] Park M (2018). Concerted action of evolutionarily ancient and novel SNARE complexes in flowering-plant cytokinesis. Dev. Cell.

[33] Adebesin F (2017). Emission of volatile organic compounds from petunia flowers is facilitated by an ABC transporter. Science.

[34] Kang J (2011). Plant ABC transporters. Arabidopsis Book.

[35] Kubo H (2018). Biosynthesis of riccionidins and marchantins is regulated by R2R3-MYB transcription factors in Marchantia polymorpha. J. Plant Res..

[36] Stahl E (1888). Pflanzen und Schnecken. Jena. Z. Med. Naturwiss.

[37] Romani F (2020). Oil body formation in *Marchantia polymorpha* is controlled by MpC1HDZ and serves as a defense against arthropod herbivores. Curr. Biol..

[38] Minamino N (2018). RAB GTPases in the basal land plant *Marchantia polymorpha*. Plant Cell Physiol..

[39] Rutherford S, Moore I (2002). The *Arabidopsis Rab* GTPase family: another enigma variation. Curr. Opin. Plant Biol..

[40] Chow CM, Neto H, Foucart C, Moore I (2008). Rab-A2 and Rab-A3 GTPases define a trans-golgi endosomal membrane domain in *Arabidopsis* that contributes substantially to the cell plate. Plant Cell.

[41] Kirchhelle C (2016). The specification of geometric edges by a plant rab GTPase is an essential cell-patterning principle during organogenesis in *Arabidopsis*. Developmental cell.

[42] Ivanov S, Harrison MJ (2014). A set of fluorescent protein-based markers expressed from constitutive and arbuscular mycorrhiza-inducible promoters to label organelles, membranes and cytoskeletal elements in *Medicago truncatula*. Plant J.: Cell Mol. Biol..

[43] Pumplin N, Zhang X, Noar RD, Harrison MJ (2012). Polar localization of a symbiosis-specific phosphate transporter is mediated by a transient reorientation of secretion. Proc. Natl Acad. Sci. USA.

[44] Agop-Nersesian C (2010). Biogenesis of the inner membrane complex is dependent on vesicular transport by the alveolate specific GTPase Rab11B. PLoS Pathog..

[45] Di Sansebastiano GP (2015). Subcellular compartmentalization in protoplasts from *Artemisia annua* cell cultures: engineering attempts using a modified SNARE protein. J. Biotechnol..

[46] Edgar RC (2004). MUSCLE: a multiple sequence alignment method with reduced time and space complexity. BMC Bioinforma..

[47] Edgar RC (2004). MUSCLE: multiple sequence alignment with high accuracy and high throughput. Nucleic Acids Res..

[48] Castresana J (2000). Selection of conserved blocks from multiple alignments for their use in phylogenetic analysis. Mol. Biol. Evol..

[49] Talavera G, Castresana J (2007). Improvement of phylogenies after removing divergent and ambiguously aligned blocks from protein sequence alignments. Syst. Biol..

[50] Guindon S (2010). New algorithms and methods to estimate maximum-likelihood phylogenies: assessing the performance of PhyML 3.0. Syst. Biol..

[51] Ishizaki K (2015). Development of gateway binary vector series with four different selection markers for the liverwort *Marchantia polymorpha*. PLoS ONE.

[52] Buschmann H, Holtmannspotter M, Borchers A, O’Donoghue MT, Zachgo S (2016). Microtubule dynamics of the centrosome-like polar organizers from the basal land plant *Marchantia polymorpha*. N. Phytologist.

[53] Otani K (2018). An evolutionarily conserved NIMA-related kinase directs rhizoid tip growth in the basal land plant *Marchantia polymorpha*. Development.

[54] Naito Y, Hino K, Bono H, Ui-Tei K (2015). CRISPRdirect: software for designing CRISPR/Cas guide RNA with reduced off-target sites. Bioinformatics.

[55] Sugano SS (2018). Efficient CRISPR/Cas9-based genome editing and its application to conditional genetic analysis in *Marchantia polymorpha*. PLoS ONE.

[56] Ishizaki K, Johzuka-Hisatomi Y, Ishida S, Iida S, Kohchi T (2013). Homologous recombination-mediated gene targeting in the liverwort *Marchantia polymorpha* L. Sci. Rep..

[57] Ishizaki K, Chiyoda S, Yamato KT, Kohchi T (2008). Agrobacterium-mediated transformation of the haploid liverwort *Marchantia polymorpha* L., an emerging model for plant biology. Plant Cell Physiol..

[58] Kubota A, Ishizaki K, Hosaka M, Kohchi T (2013). Efficient Agrobacterium-mediated transformation of the liverwort Marchantia polymorpha using regenerating thalli. Biosci., Biotechnol., Biochem..

[59] Minamino N (2017). Dynamic reorganization of the endomembrane system during spermatogenesis in *Marchantia polymorpha*. J. Plant Res..

[60] Honkanen S (2016). The mechanism forming the cell surface of tip-growing rooting cells is conserved among land plants. Curr. Biol..

[61] Liu YG, Mitsukawa N, Oosumi T, Whittier RF (1995). Efficient isolation and mapping of *Arabidopsis-Thaliana* T-DNA insert junctions by thermal asymmetric interlaced Pcr. Plant J..

[62] Bray NL, Pimentel H, Melsted P, Pachter L (2016). Near-optimal probabilistic RNA-seq quantification. Nat. Biotechnol..

[63] Robinson MD, McCarthy DJ, Smyth GK (2010). edgeR: a Bioconductor package for differential expression analysis of digital gene expression data. Bioinformatics.

[64] Saint-Marcoux D, Proust H, Dolan L, Langdale JA (2015). Identification of reference genes for real-time quantitative PCR experiments in the liverwort *Marchantia polymorpha*. PLoS ONE.

[65] Nakazaki A (2019). Leaf endoplasmic reticulum bodies identified in *Arabidopsis Rosette* leaves are involved in defense against herbivory. Plant Physiol..

[66] Bowman JL (2016). The naming of names: guidelines for gene nomenclature in marchantia. Plant cell Physiol..

[67] Montgomery, S. A. et al. Chromatin organization in early land plants reveals an ancestral association between H3K27me3, transposons, and constitutive heterochromatin. *Curr. Biol*. 10.1016/j.cub.2019.12.015 (2020). 10.1016/j.cub.2019.12.015 PMC7209395 32004456

