# Supplementary material for: The liverwort oil body is formed by redirection of the secretory pathway
Source: Nat Commun. 2020 Dec 1;11:6152. doi: 10.1038/s41467-020-19978-1 (PMC7708844; doi:10.1038/s41467-020-19978-1)
Supplement: Supplementary file 1 — Supplementary Information [file 41467_2020_19978_MOESM1_ESM.pdf]

Supplementary Information for

The liverwort oil body is formed by redirection of the secretory pathway

Takehiko Kanazawa, Hatsune Morinaka, Kazuo Ebine, Takashi L. Shimada, Sakiko Ishida, Naoki Minamino, Katsushi Yamaguchi, Shuji Shigenobu, Takayuki Kohchi, Akihiko Nakano, Takashi Ueda.

Correspondence to: tueda@nibb.ac.jp

This PDF file includes:

Supplementary Figures 1-6

Other Supplementary Materials for this manuscript include the following:

Supplementary Movies 1 & 2
Supplementary Data 1-5

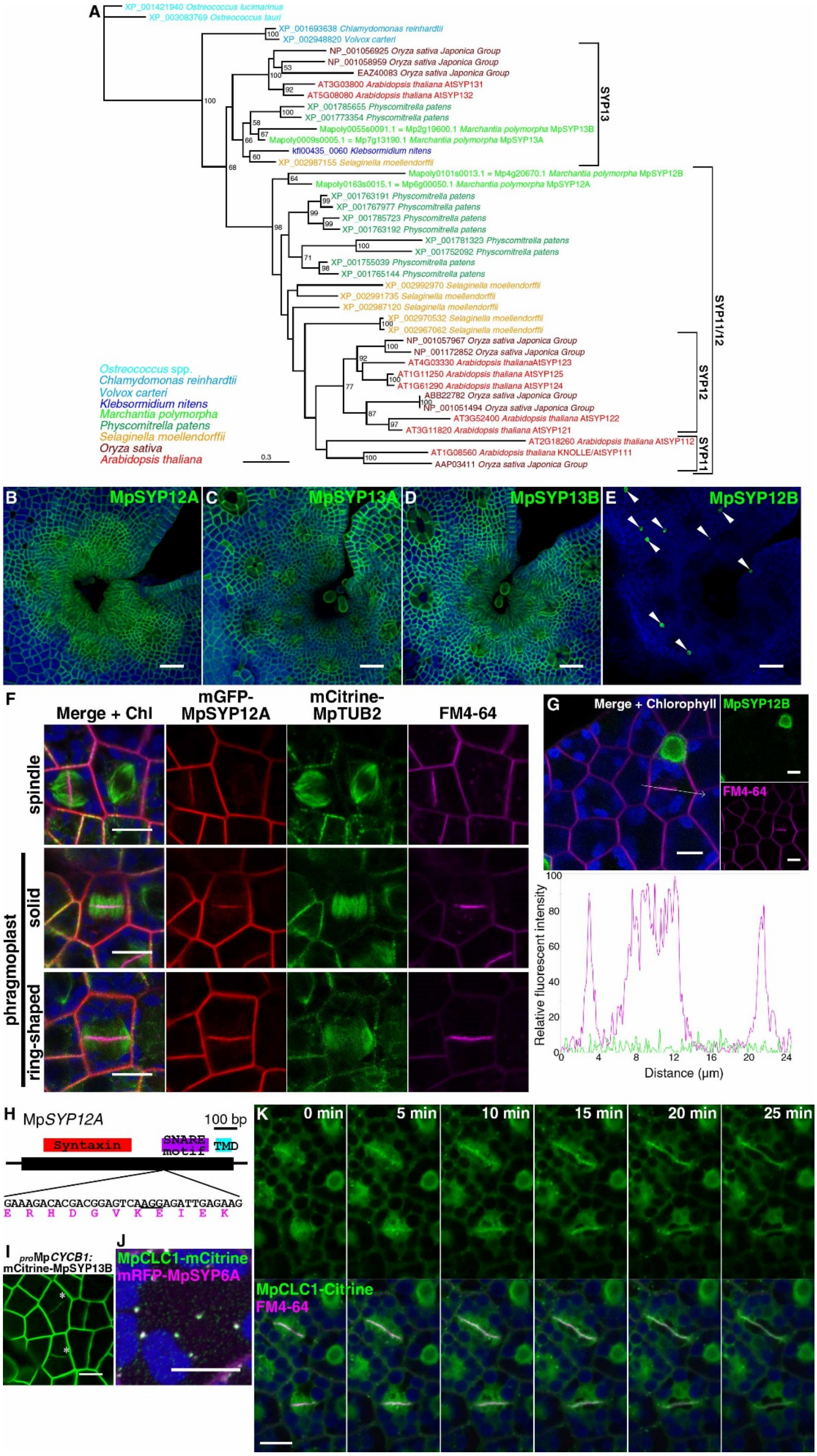
A
B MpSYP12A
C MpSYP13A
D MpSYP13B
E MpSYP12B
F
Merge + Chl
mGFP-MpSYP12A
mCitrine-MpTUB2
FM4-64
spindle
phragmoplast
solid
ring-shaped
G Merge + Chlorophyll
MpSYP12B
FM4-64
Relative fluorescent intensity
Distance (μm)
H MpSYP12A
100 bp
Syntaxin
SNARE motif
TMD
GAAAGACACGACGGAGTCAAGGAGATTGAGAAG
E R H D G V K E I E K
I proMpCYCB1: mCitrine-MpSYP13B
J MpCLC1-mCitrine
mRFP-MpSYP6A
K 0 min
5 min
10 min
15 min
20 min
25 min
MpCLC1-Citrine
FM4-64
SYP13
SYP11/12
SYP12
SYP11
Ostreococcus spp.
Chlamydomonas reinhardtii
Volvox carteri
Klebsormidium nitens
Marchantia polymorpha
Physcomitrella patens
Selaginella moellendorffii
Oryza sativa
Arabidopsis thaliana
0.3

Supplementary Figure 1. Characterization of SYP1 member and clathrin light chain localization during cytokinesis in Marchantia thalli.

A A maximum likelihood phylogenetic tree of SYP1 members in green plants. Although the tree is unrooted, the proteins from Chlorophytes (*Ostreococcus* spp., *Chlamydomonas reinhardtii*, and *Volvox carteri*) are the outgroup to those of streptophytes. The branch lengths are proportional to the estimated number of substitutions per site. Bootstrap probability is indicated as a percentage on each branch with at least 50% support. **B–E** Maximum intensity projection images of thalli expressing mCitrine-MpSYP12A (B), mCitrine-MpSYP13A (C), mCitrine-MpSYP13B (D), or mCitrine-MpSYP12B (E). White arrowheads indicate mCitrine-MpSYP12B-expressing cells. **F** Single confocal images of thallus cells expressing mCitrine-MpTUB2 and mGFP-MpSYP12A under the regulation of the Mp*SYP2* and Mp*SYP12A* promoters, respectively, stained with FM-4-64. In mitotic cells, spindles and phragmoplasts (solid and ring-shaped phases) were labelled by mCitrine-MpTUB2, and endocytosed FM4-64 accumulated at forming cell plates. **G** Single confocal images of thallus cells expressing mCitrine-MpSYP12B stained with FM4-64. The line graph indicates the relative fluorescence intensity along the white arrow. Relative fluorescent intensity of mCitrine to fluorescence on the oil body membrane is shown for mCitrine fluorescence. **H** The gene model and sequences of MpSYP12A. The syntaxin domain, SNARE motif, and transmembrane domain (TMD) are shown above the gene model. Black and magenta letters indicate genome and translated amino acid sequences. The protospacer adjacent motif (PAM) sequence for gRNA is underlined. **I** Expression by the Mp*CYCB1* promoter results in accumulation of MpSYP13B at the forming cell plate. A single confocal image of dividing thallus cells expressing mCitrine-MpSYP13B are shown. Asterisks indicate forming cell plates. **J** A maximum intensity projection image of a thallus cell expressing MpCLC1-mCitrine and mRFP-MpSYP6A under the regulation of their own regulatory elements and the Mp*EF1α* promoter, respectively. **K** Time-lapse single confocal images of thallus cells expressing MpCLC1-Citrine under the regulation of the Mp*EF1α* promoter (upper panels) and merged images with FM4-64 signals. Autofluorescence from chlorophyll is pseudo-coloured in blue. Bars = 50 μm in (B - E) and 10 μm in (F), (G), (I), (J), and (K).

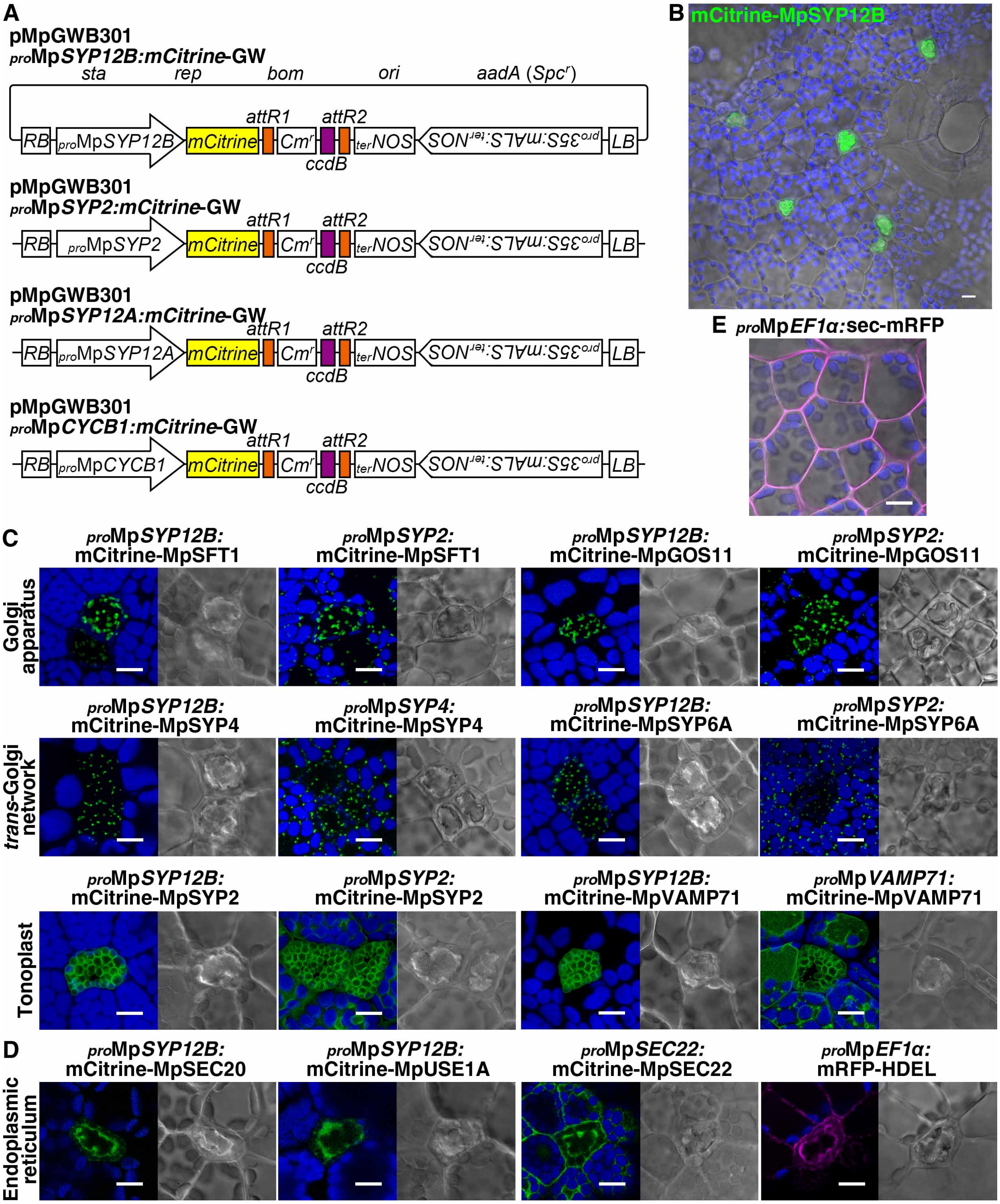

Supplementary Figure 2. Subcellular localization of organelle markers in oil body cells.
A Gateway constructs used in this study. pMpGWB301 $_{pro}$Mp*SYP2:*mCitrine-GW was previously prepared [59]. **B** A maximum intensity projection image of thallus cells including oil body cells expressing mCitrine-MpSYP12B under its own regulatory elements. **C** Maximum intensity projection images of thallus cells including the oil body cell expressing mCitrine-

fused markers for the Golgi apparatus (MpSFT1 and MpGOS11), *trans*-Golgi network (MpSYP4 and MpSYP6A), and tonoplast (MpSYP2 and MpVAMP71). The organelle markers were expressed under the Mp*SYP12B*, Mp*SYP2*, and/or own promoters. Bright field images are also shown. **D** Single confocal images of thallus cells including the oil body cell expressing endoplasmic reticulum markers. mCitrine-MpSEC20 and mCitrine-MpUSE1A were expressed under the Mp*SYP12B* promoter. mCitrine-MpSEC22 and mRFP-HDEL were expressed using their own regulatory elements and the Mp*EF1α* promoter, respectively. **E** A single confocal image of thallus cells expressing sec-mRFP expressed under the Mp*EF1α* promoter. Green, magenta, and blue pseudo-colours indicate fluorescence from mCitrine, mRFP, and chlorophyll, respectively. Bars = 10 μm.

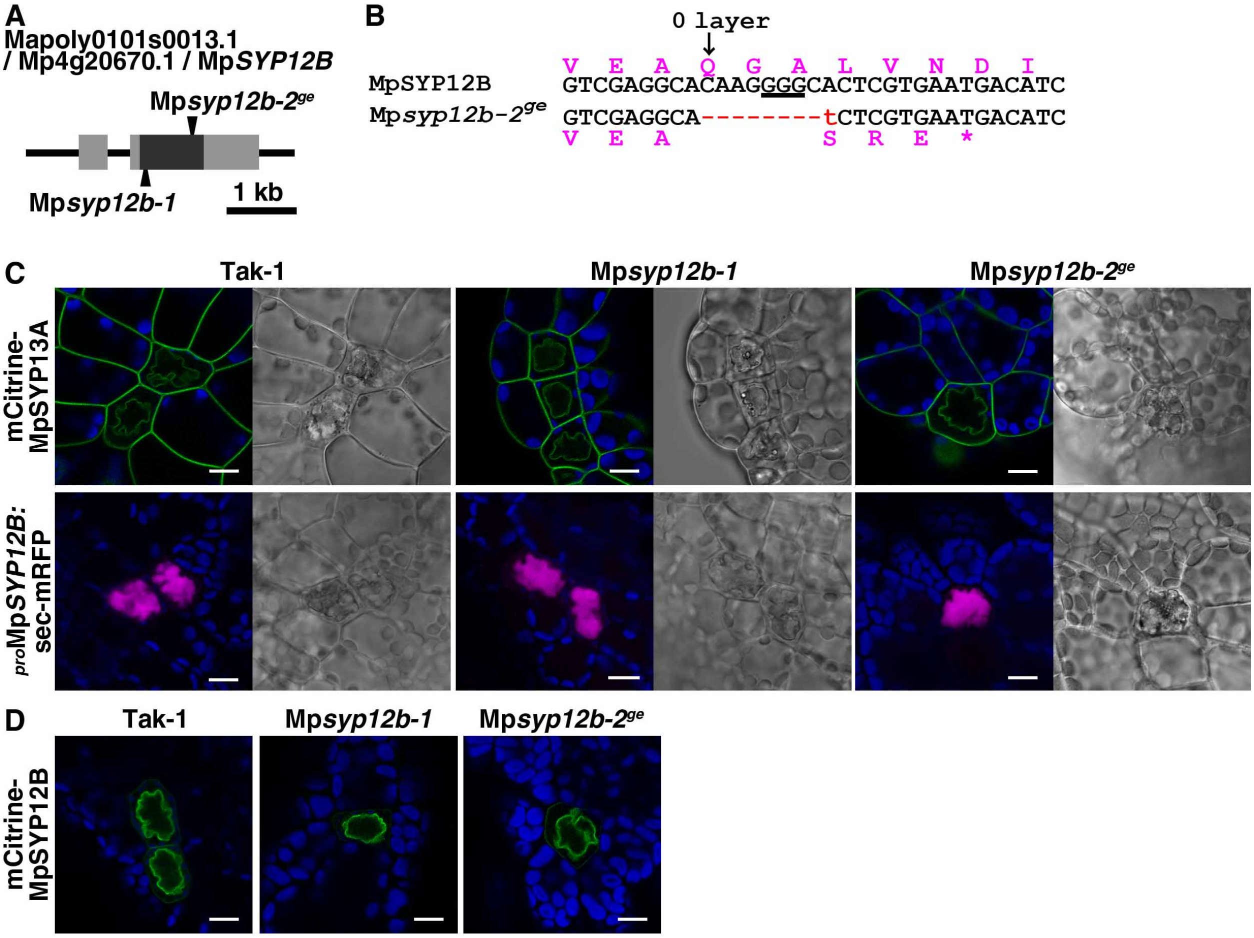

Supplementary Figure 3. Phenotype of Mp*syp12b* mutants.

A Schematic representation of the Mp*SYP12B* gene structure. Gray and black boxes indicate untranslated and coding regions, respectively, and arrowheads indicate mutation sites in two independently generated mutants. The Mp*syp12b-1* mutant was generated by a homologous recombination-mediated gene targeting method [56]. **B** The genome (black letters) and translated amino acid (magenta letters) sequences of Mp*SYP12B* and the Mp*syp12b-2^{ge}* mutant, with the red letters indicating the mutated region. The PAM sequence for gRNA is underlined. The 0 layer at the centre of the SNARE motif is shown. **C** Single confocal images of thallus cells including the oil body cell expressing mCitrine-MpSYP13A under its own regulatory elements, or sec-mRFP under the Mp*SYP12B* promoter in Tak-1, Mp*syp12b-1*, and Mp*syp12b-2^{ge}* lines. Bright field images are also shown. **D** Single confocal images of thallus cells including the oil body cell expressing mCitrine-MpSYP12B under its own regulatory elements in Tak-1 and Mp*syp12b* mutants. Green, magenta, and blue pseudo-colours indicate fluorescence from mCitrine, mRFP, and chlorophyll, respectively. Bars = 10 μm.

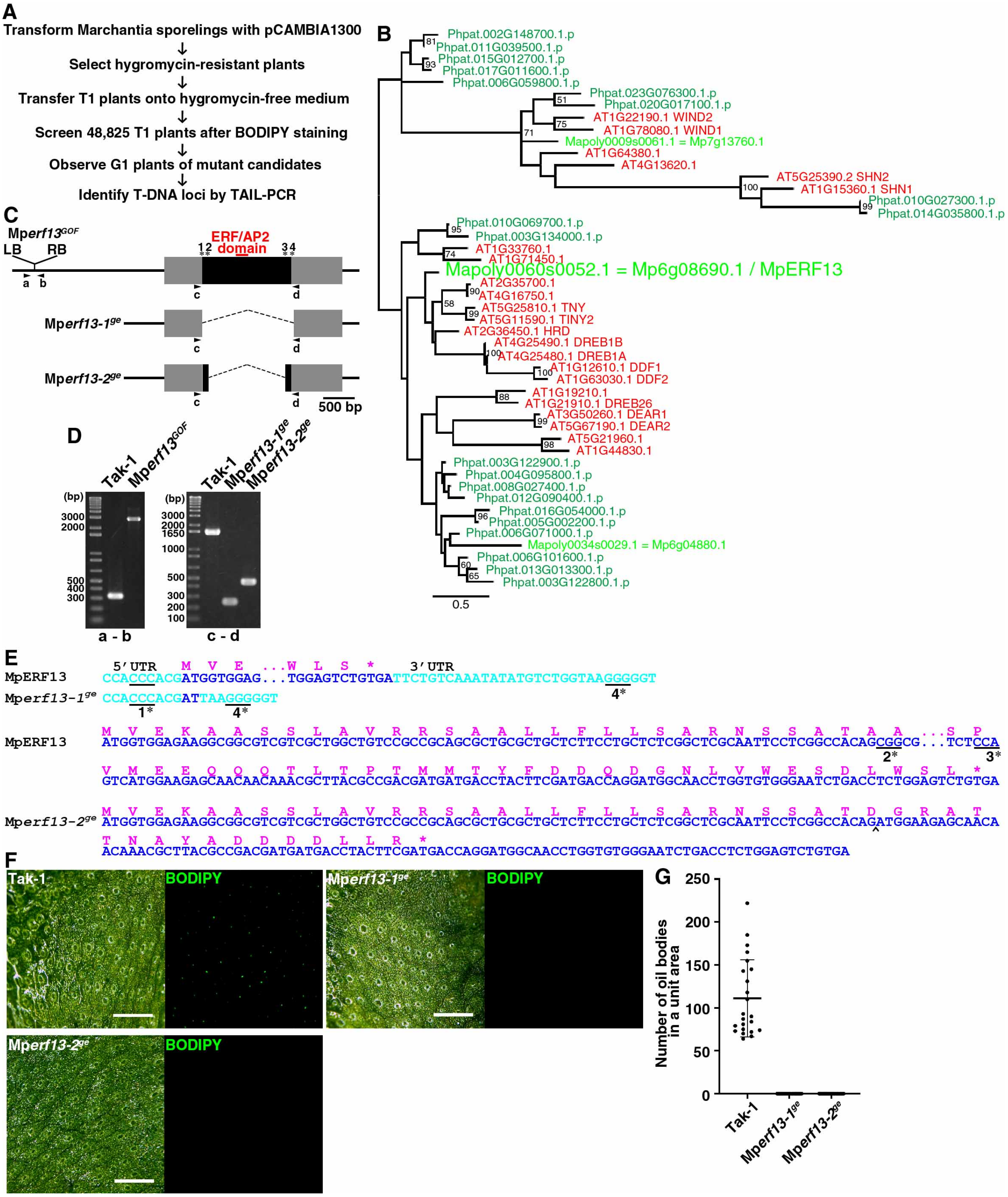

Supplementary Figure 4. Screening and generation of Mp*erf13* mutants.

A The flow chart of the screen for mutants defective in oil body formation from T-DNA-insertion lines. **B** A maximum likelihood phylogenetic tree of proteins with one ERF/AP2 domain. The colour code is shown in Supplementary Figure 1A. The branch lengths are proportional to the estimated number of substitutions per site. Bootstrap probability is indicated as a percentage on each branch with at least 50% support. A more detailed tree was

presented previously [27]. **C** Schematic representation of the Mp*ERF13* gene structure and mutations generated in this study. Gray and black boxes indicate the UTR and coding sequences, respectively. Asterisks with numbers indicate sites of designed gRNA to generate Mp*erf13-1*^{ge} (*1 and *4) and Mp*erf13-2*^{ge} (*2 and *3). **D** PCR-based genotyping of Tak-1, Mp*erf13*^{GOF}, Mp*erf13-1*^{ge}, and Mp*erf13-2*^{ge}. The combinations and annealing sites of primers (a to d) are shown in (C). **E** The genomic and predicted amino acid sequences of the Mp*ERF13* locus in Tak-1 and Mp*erf13* mutants. Light blue, dark blue, and magenta letters indicate UTR, coding, and predicted amino acid sequences, respectively. The PAM sequences for gRNAs are underlined. The caret indicates the indel site. **F** Fluorescent and bright-field images of BODIPY-stained three-week-old thalli of Tak-1, Mp*erf13-1*^{ge}, and Mp*erf13-2*^{ge}. Bars = 0.5 mm. **G** The number of oil bodies visualized with BODIPY in a unit area (2.0 mm × 2.0 mm). Bars indicate means ± s.d. Statistical analyses between Tak-1 and each genotype were conducted using a two-tailed Welch's *t*-test. Sample numbers were 23 thalli for Tak-1, 24 for Mp*erf13-1*^{ge}, and 26 for Mp*erf13-2*^{ge}. *p*-values are 5.14×10^{-11} for Mp*erf13-1*^{ge} and 5.14×10^{-11} for Mp*erf13-2*^{ge}.

A

Gene ID Ver3.1	Gene ID Ver5.1	Description	logFC (Mp*erf13*^{GOF}/Tak-1)	logFC (Mp*erf13-1*^{ge}/Tak-1)
Mapoly0083s0014	Mp8g13070	ABC transporter	4.273	-3.722
Mapoly2351s0001	Mp2g20510	Cytochrome P450	6.795	-5.717
Mapoly0119s0008	Mp4g14860	LEA protein	5.045	-3.099
Mapoly0101s0013	Mp4g20670	MpSYP12B	4.374	-5.777
Mapoly0006s0226	Mp3g07510	MpMYB02	3.053	-7.208
Mapoly0060s0052	Mp6g08690	MpERF13	4.734	-5.205
Mapoly0110s0019	Mp8g13380	Cellulase	4.005	-6.537
Mapoly0005s0135	Mp1g04730	Lipid transfer	4.454	-4.903
Mapoly0081s0021	Mp8g05200	Receptor-like kinase	2.813	-4.406
Mapoly0060s0009	Mp6g09100	Ankyrin repeats	4.243	-5.086
Mapoly0207s0009	Mp2g24710	Tomosyn12	3.698	-5.704

B

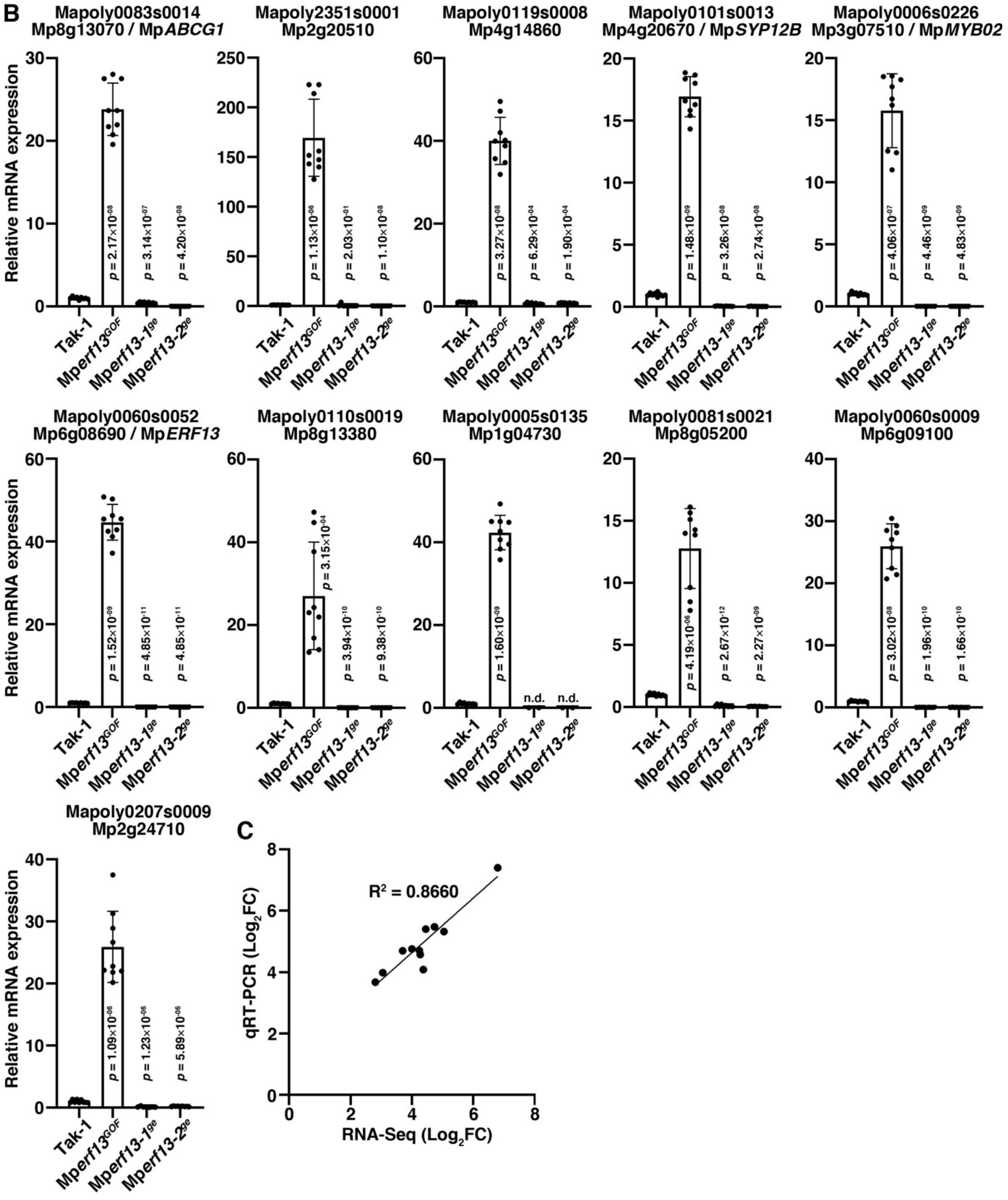

Supplementary Figure 5. Gene expression in Mp*erf13* mutants.

A List of selected differentially expressed genes (DEGs) for qRT-PCR analysis. The full list of DEGs are shown as Supplementary Table S1. **B** The relative mRNA expression of 11 DEGs in Tak-1, Mp*erf13*^{GOF}, Mp*erf13-1*^{ge}, and Mp*erf13-2*^{ge} measured by qRT-PCR. Mp*APT* was used as an internal reference. Error bars represent ± s.d. Three biological replicates were prepared and three technical replicates were performed for each gene. Statistical comparison between Tak-1 and each genotype was conducted with a two-tailed Welch's *t*-test. The *p* values are indicated in the graphs. **C** Correlation of the expression levels between the RNA-Seq analysis and qRT-PCR experiment. Both the x- and y- axes are shown in a $\log_2$FC scale. R^2 indicates the correlation coefficient.

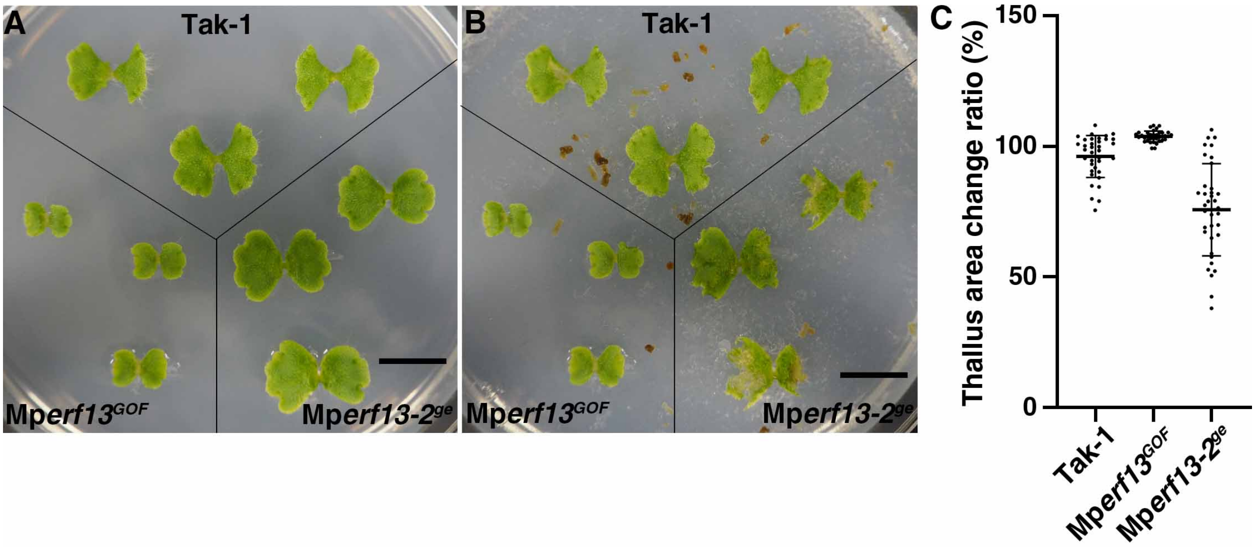

Supplementary Figure 6. Pill bug feeding assay.

A and B Ten-day-old thalli of Tak-1, Mp*erf13*^{GOF} and Mp*erf13-2*^{ge} (**A**) were co-cultivated with starved pill bugs for 24 hr (**B**). Bars = 1 cm. **C** Ratios of thallus area before and after co-cultivation with pill bugs were calculated (n = 36 thalli for each genotype). Bars indicate means ± s.d. Statistical analyses between Tak-1 and each genotype were conducted with a two-tailed Welch's *t*-test. The *p*-values were 2.02×10^{-6} for Mp*erf13*^{GOF} and 7.93×10^{-8} for Mp*erf13-2*^{ge}.
