# Supplementary material for: The liverwort oil body is formed by redirection of the secretory pathway
Source: Nat Commun. 2020 Dec 1;11:6152. doi: 10.1038/s41467-020-19978-1 (PMC7708844; doi:10.1038/s41467-020-19978-1)
Supplement: Supplementary file 2 — Descriptions of Additional Supplementary Files [file 41467_2020_19978_MOESM2_ESM.pdf]

Descriptions of Additional Supplementary Files

Supplementary Data 1

Description: List of DEGs in Mperf13 mutants identified in RNA-Seq analysis.

Supplementary Data 2

Description: List of primers used in this study.

Supplementary Data 3

Description: Reactions of TAIL-PCR.

Supplementary Data 4

Description: List of accession numbers of genes used in this study.

Supplementary Data 5

Description: List of transgenic plants generated and cell numbers observed in this study.

Supplementary Movie 1

Description: Formation of clathrin-coated vesicles at the oil body membrane. A time-lapse movie of an oil body cell expressing MpCLC1-Citrine. Green and blue pseudo-colours represent fluorescence from Citrine and chlorophyll, respectively. 32× real time. Bar = 5 µm.

Supplementary Movie 2

Description: Expression of MpSYP12B in a Marchantia thallus A five-day-old thallus expressing 2×YFP driven by the MpSYP12B promoter observed by light sheet microscopy. Green and blue pseudo-colours represent fluorescence from Citrine and chlorophyll, respectively. Bar = 400 µm.
